# The DnaK Chaperone Uses Different Mechanisms To Promote and Inhibit Replication of *Vibrio cholerae* Chromosome 2

**DOI:** 10.1128/mBio.00427-17

**Published:** 2017-04-18

**Authors:** Jyoti K. Jha, Mi Li, Rodolfo Ghirlando, Lisa M. Miller Jenkins, Alexander Wlodawer, Dhruba Chattoraj

**Affiliations:** a Laboratory of Biochemistry and Molecular Biology, CCR, NCI, NIH, Bethesda, Maryland, USA; b Macromolecular Crystallography Laboratory, NCI, Frederick, Maryland, USA; c Basic Science Program, Leidos Biomedical Research, Frederick National Laboratory for Cancer Research, Frederick, Maryland, USA; d Laboratory of Molecular Biology, NIDDK, NIH, Bethesda, Maryland, USA; e Laboratory of Cell Biology, CCR, NCI, NIH, Bethesda, Maryland, USA; University of Minnesota Medical School

**Keywords:** chromosome replication, DNA-protein interactions, DnaK chaperone, initiator remodeling, initiator structure, *Vibrio cholerae*

## Abstract

Replication of *Vibrio cholerae* chromosome 2 (Chr2) depends on molecular chaperone DnaK to facilitate binding of the initiator (RctB) to the replication origin. The binding occurs at two kinds of site, 12-mers and 39-mers, which promote and inhibit replication, respectively. Here we show that DnaK employs different mechanisms to enhance the two kinds of binding. We found that mutations in *rctB* that reduce DnaK binding also reduce 12-mer binding and initiation. The initiation defect is suppressed by second-site mutations that increase 12-mer binding only marginally. Instead, they reduce replication inhibitory mechanisms: RctB dimerization and 39-mer binding. One suppressing change was in a dimerization domain which is folded similarly to the initiator of an iteron plasmid—the presumed progenitor of Chr2. In plasmids, DnaK promotes initiation by reducing dimerization. A different mutation was in the 39-mer binding domain of RctB and inactivated it, indicating an alternative suppression mechanism. Paradoxically, although DnaK increases 39-mer binding, the increase was also achieved by inactivating the DnaK binding site of RctB. This result suggests that the site inhibits the 39-mer binding domain (via autoinhibition) when prevented from binding DnaK. Taken together, our results reveal an important feature of the transition from plasmid to chromosome: the Chr2 initiator retains the plasmid-like dimerization domain and its control by chaperones but uses the chaperones in an unprecedented way to control the inhibitory 39-mer binding.

## IMPORTANCE

The capacity of proteins to undergo remodeling provides opportunities to control their function. However, remodeling remains a poorly understood aspect of the structure-function paradigm due to its dynamic nature. Here we have studied remodeling of the initiator of replication of *Vibrio cholerae* Chr2 by the molecular chaperone, DnaK. We show that DnaK binds to a site on the Chr2 initiator (RctB) that promotes initiation by reducing the initiator’s propensity to dimerize. Dimerization of the initiator of the putative plasmid progenitor of Chr2 is also reduced by DnaK, which promotes initiation. Paradoxically, the DnaK binding also promotes replication inhibition by reducing an autoinhibitory activity of RctB. In the plasmid-to-chromosome transition, it appears that the initiator has acquired an autoinhibitory activity and along with it a new chaperone activity that apparently helps to control replication inhibition independently of replication promotion.

## INTRODUCTION

The bacterial chaperone DnaK and its eukaryotic homolog Hsp70 are highly conserved proteins. They affect a sizable fraction of the proteome by modulating folding, remodeling, assembly and disassembly, and disaggregation and degradation of proteins, thereby affecting a variety of cellular functions (1). The mechanisms of chaperone action are varied, and their determination is of significant interest, particularly since improper chaperone function can lead to many diseases (2).

The DnaK chaperone plays major roles in DNA replication. DnaK was discovered together with cochaperones DnaJ and GrpE (the DnaK system) in bacteria as factors required in bacteriophage lambda replication (3). The system performs an essential function in replication by helping to load the replicative helicase, DnaB, onto an open strand of the replication origin (4). The helicase is brought to the replication origin through binding of lambda protein P. However, in the P-DnaB complex, the helicase and single-stranded DNA binding activities of DnaB are suppressed. The chaperones break the P-DnaB contact that activates the helicase.

The same chaperones also promote replication of a family of plasmids characterized by the presence of repeated initiator binding sites (iterons) in their origin, but they do so by a different mechanism. In iteron plasmids, the chaperones promote initiator binding to iterons (5). Plasmid initiators can dimerize but bind to iterons as monomers that initiate replication. The chaperones help in monomerization and thereby increase monomer binding to the origin. It was also proposed that dimer dissociation *per se* is not sufficient and that monomers need to be remodeled to promote iteron binding (6). The remodeling was later evident from the comparison of structures of initiator monomers and dimers (7, 8). In addition to iteron binding, the chaperones promote plasmid replication by reducing the activity of an inhibitory regulatory mechanism called “handcuffing.” Although the dimers are inactive in iteron binding, they are believed to bridge monomer-bound iterons of sister origins (handcuffing), which inhibits further firing of the origins (9, 10). The reduction in dimerization can help free the origins for the next round of replication (11). Thus, the chaperones could facilitate replication both by promoting a positive function (initiator binding) and by reducing an inhibitory function (handcuffing).

Chaperones also participate in chromosomal replication, with their role well documented in *Caulobacter crescentus* (12). There they help to accumulate sufficient initiator DnaA to allow initiation. The accumulation comes from restraining the synthesis of a protease, Lon, which otherwise would degrade DnaA. Another protease, ClpAP, also serves as an auxiliary enzyme to control the level of DnaA (13). DnaK is normally not required for *Escherichia coli* replication, although it can disaggregate DnaA *in vitro* and activate mutant forms of DnaA for initiation (14).

We investigated the role of DnaK in DNA replication using *Vibrio cholerae*, a bacterium with two chromosomes (Chr1 and Chr2). *V. cholerae* is currently the leading exemplar of bacterial species with divided genomes used for studying chromosome maintenance. Chr1 replication is regulated by DnaA, as in *E. coli*, and Chr2 replication is regulated by RctB, a Chr2-specific initiator (15). The replication origin of Chr2 (*ori2*) is organized similarly to that of the iteron plasmids, and the chromosome is believed to have originated from such a plasmid (16). As in iteron plasmids, replication of *ori2*-driven plasmids (mini-Chr2), as well as the *in vitro* binding of RctB to Chr2 sites that correspond to iterons (called 12-mers), is severely defective in an *E. coli* Δ*dnaKJ* host (17).

The initiation of Chr2 replication, however, is more complex than that of iteron plasmids. Chr2 replicates at a particular time of the cell cycle, whereas there is generally no fixed time for plasmid replication (16). The initiator binding to Chr2 origin is also more complex. In addition to 12-mers, RctB binds to a second kind of site, the 39-mer, that inhibits Chr2 replication (18). In other words, the promotion and inhibition of replication are mediated by 12-mers and 39-mers, respectively, whereas both functions are mediated by iterons in plasmids. Another important difference is that in addition to monomers, dimers bind to 12-mers *in vitro*; the 39-mers bind monomers only (19). In all cases, binding is promoted by DnaJK. In two respects, the initiator binding in Chr2 is exceptional. First, DnaJK promotes dimer-*ori2* binding, whereas dimers do not bind iterons in plasmids and the dimerization is reduced by chaperones (5). Second, in the replication systems discussed above, the chaperones play only positive roles. The apparently paradoxical facilitation of both replication-promoting 12-mer binding and replication-inhibiting 39-mer binding by chaperones is a distinguishing feature of Chr2 replication control. The understanding of the regulation of initiator binding to *ori2* is central to our understanding of the timing and frequency of replication initiation in the cell cycle, which remains unclear even in well-studied bacteria (16).

Here we investigate the role that DnaK plays in promoting Chr2 replication initiation and compare our findings with the known role of DnaK in plasmids of the class to which the presumed progenitor of Chr2 belongs. Our work shows that distinct mechanisms promote RctB binding to 12-mers and 39-mers by DnaK (17). This was evident from the finding that DnaK interacts with a specific site of RctB (named the K-interaction site [or “K-I site”]) and from subsequently showing that mutating this site reduces RctB-DnaK interaction and abrogates 12-mer binding but makes RctB-39-mer binding independent of DnaK. Participation of DnaK in 12-mer binding appears to follow the plasmid paradigm since we showed that RctB contains a dimerization domain which is folded similarly to that of initiators of iteron plasmids. This finding corroborates the presumed evolutionary link between the two replicons. A similar link was also reported in a recent study (20). Reduction in RctB dimerization could promote replication, but chaperones were still required. This indicates not only that dimerization is an inhibitory mechanism but also that remodeling of monomers is an obligatory role of the chaperones. The relief from dependence on DnaK for 39-mer binding by the inactivation of the K-I site suggests that the site is inhibitory for 39-mer binding and that the inhibition is overcome by DnaK binding, a role of the chaperone not previously described in the field of DNA replication.

## RESULTS

### Identification of a site in RctB that interacts with DnaK (the K-I site).

The DnaK chaperone interacts with many client proteins, but what it recognizes on the client is not easily defined; only preferences for some specific residues have been reported (21–23). Given the significant role of DnaK in promoting RctB binding to both 12-mers and 39-mers *in vitro* as well as replication of mini-Chr2 in *E. coli* (17), we set out to determine the identity of the K-I site of RctB.

We initially determined interaction of DnaK with maltose binding protein (MBP)-tagged RctB (to enhance its solubility [24]) and untagged RctB by coimmunoprecipitation (Co-IP) and found that the MBP tag does not interfere with binding to DnaK (see Fig. S1A in the supplemental material). We also found that a previously constructed mutant of RctB (MBP-N450) in which the C-terminal 158 residues were deleted also interacted with DnaK (see Fig. S1B in the supplemental material) (19).

10.1128/mBio.00427-17.2 FIG S1  Identification of an RctB region important for interaction with DnaK *in vitro* and *in vivo*. Download FIG S1, DOCX file, 0.6 MB. Copyright © 2017 Jha et al. 2017 Jha et al. This content is distributed under the terms of the Creative Commons Attribution 4.0 International license.

We next made a series of progressively larger N-terminal deletions (ΔN10, ΔN20, ΔN50, ΔN100, ΔN200, and ΔN400) of RctB and tested them for interaction with DnaK (see Fig. S1C in the supplemental material). There was a significant decrease in the amount of DnaK coprecipitated with ΔN200 compared to that coprecipitated with ΔN100, indicating that a K-I site is present in residue interval 101 to 200 of RctB.

We confirmed our *in vitro* results using the yeast two-hybrid (Y2H) assay (25) and a bacterial two-hybrid assay (26). In both the assays, wild-type (WT) RctB and ΔN100 RctB, but not ΔN200 RctB, scored positive (see Fig. S1D and E in the supplemental material).

### Residues in interval 150 to 163 of RctB are important for interaction with DnaK.

**FIG 1:**
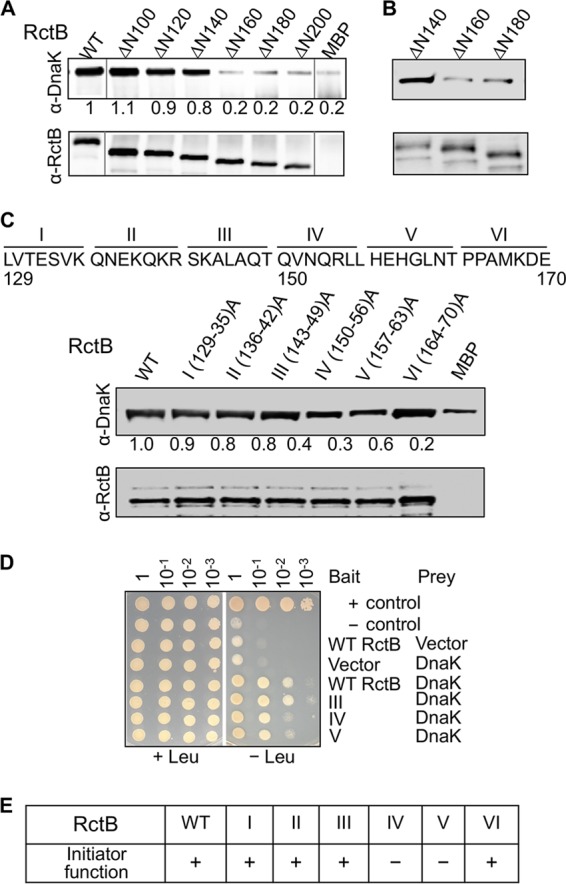
RctB residues 150 to 163 are important for interactions with DnaK and initiator activity. (A) Interaction of DnaK and MBP-tagged RctB assayed by Co-IP. RctB and its deletion derivatives, identified at the top of the figure, were N-terminally tagged with MBP. The proteins were treated with DnaKJ and ATP and subsequently with amylose magnetic beads. The bead-bound proteins were analyzed by Western blotting using antibodies against RctB (α-RctB) and DnaK (α-DnaK). The MBP tag alone was used as a negative control, and it shows some binding, apparently reflecting the promiscuous nature of DnaK binding (22). Note that some lanes were spliced out from the gel picture because the data were present in Fig. S1C in the supplemental material. The spliced junctions are marked with gray vertical lines next to WT lane and before the MBP lane. (B) Same as panel A, except that instead of purified RctB, extracts of cells expressing RctB mutant ΔN140, ΔN160, or ΔN180 were used. (C) Same as panel A, except that RctB had alanine substitutions. RctB residues 129 to 170 were converted seven at a time to alanine to generate mutants I to VI. (D) Interactions of RctB WT and mutants III to V with DnaK determined by the yeast two-hybrid assay. Plasmids pSH17-34 and pRFHM1 were used as positive and negative controls, respectively. (E) Initiator activity of RctB. The proteins were all untagged. Positive initiator activity (+) indicates that cells with a RctB source could be transformed with a mini-Chr2 (pJJ114). Negative initiator activity (–) is indicated if no (<10^−3^) transformants were seen upon selection for the mini-Chr2.

We made incremental 20-residue deletions (ΔN120, ΔN140, ΔN160, and ΔN180) in residue interval 101 to 200 to further define the K-I site (Fig. 1A). Our Co-IP experiments indicated that the important residues are present beyond residue 140. We also found that more DnaK precipitated from total lysates of cells expressing ΔN140 than from cells expressing ΔN160 or ΔN180 (Fig. 1B).

To further define the K-I site, we focused on the interval of 130 to 170 of RctB and changed seven residues at a time to alanine. This resulted in six mutants (mutants I to VI), covering residues 130 to 136, 137 to 142, 143 to 149, 150 to 156, 157 to 163, and 164 to 170, respectively (Fig. 1C). Of these, mutants IV and V showed the most visible defect, indicating that interval 150 to 163 of RctB is the most important for DnaK binding. We confirmed this result using the Y2H assay, which showed that mutants IV and V, but not mutant III, are defective in interactions with DnaK (Fig. 1D).

The 150-to-163 stretch, QVNQRLLHEHGLNT, although enriched in leucine residues, is not predicted by the algorithm deduced by Rüdiger et al. (22) to be a DnaK binding site mainly because of the presence of the glutamic acid. In RepA, the initiator of the iteron-plasmid P1, important residues for DnaK recognition are in the sequence ^36^RLGVFVPKPSKSKG^49 ^(21). This sequence is easily predicted to be a DnaK binding site: the combined energy value ΔΔ*G*_*K*_ for the MR-LGVGF-PK sequence of RepA is −8.81 in contrast to −0.93 for the QR-LLHEH-GL sequence of RctB (22). The recognition code of DnaK thus remains challenging to define.

### Initiator and DNA binding activities of alanine mutants of RctB.

We next tested the initiator function of the six alanine mutants of RctB. We used an *E. coli* strain where replication of a mini-Chr2 depends on the supply of RctB in *trans*. We found that cells supplying RctB mutants IV and V could not be transformed with a mini-Chr2 (Fig. 1E). This was not due to instability of the RctB derivatives, as the level of expression of the mutants was comparable to that of the WT (see Fig. S2A in the supplemental material). This result suggests that the K-I site is also important for the initiator function of RctB.

10.1128/mBio.00427-17.3 FIG S2  Expression levels of RctB mutants determined by Western blotting. Download FIG S2, DOCX file, 0.3 MB. Copyright © 2017 Jha et al. 2017 Jha et al. This content is distributed under the terms of the Creative Commons Attribution 4.0 International license.

**FIG 2:**
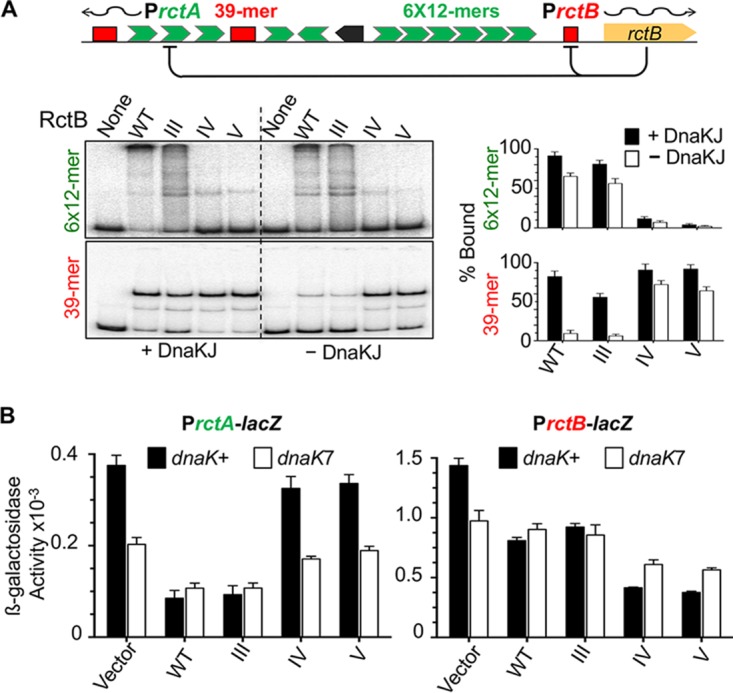
DNA binding activities of alanine mutants III to V *in vitro* and *in vivo*. (A) DNA binding of RctB determined by EMSA. The DNA fragment carried either the array of six 12-mer sites (6×12-mer) or a 39-mer site as identified in the schematic map of Chr2 origin. The fragments had 55 bp of vector sequences at both flanks of the sites. RctB, pretreated with chaperones DnaKJ (63) or not (black and white bars, respectively), was reacted with DNA at fixed concentrations (~15 nM and 1 nM, respectively) in all cases. The autoradiograms were quantified for the percentage of fragments bound by RctB, and the values are shown as bar diagrams. The solid bars represent mean levels of binding from three repeat experiments and the error bars 1 standard deviation of the mean (here and elsewhere). (B) RctB binding to 12-mers and 39-mers *in vivo* using a promoter repression assay. The origin of Chr2 has two promoters, P*rctA* and P*rctB*, that have overlapping 12-mer and 39-mer sites, respectively, which are repressed in the presence of RctB (origin diagram in panel A). The activities of the promoters were measured after fusing them to a promoter-less *lacZ* gene and measuring β-galactosidase activity. RctB proteins were same as those described for panel A, except that they were left untagged and expressed from a constitutive promoter. The vector carried the same promoter but no *rctB* gene. The β-galactosidase activities in *dnaK*^*+*^ and *dnaK7* hosts are represented by black and white bars, respectively. The activities were determined from three biological replicates. The level of RctB expression was monitored by Western blotting (Fig. S2A in the supplemental material).

RctB must bind to 12-mers to initiate replication (27). Therefore, we also tested the alanine mutants for DNA binding. In contrast to the WT and mutant III, mutants IV and V showed almost no binding to 12-mers (Fig. 2A). The binding of mutants IV and V also did not respond to the presence of DnaKJ, as would be expected if the mutants have lost their ability to interact with DnaK. These results are consistent with the requirement of region 150 to 163 for 12-mer binding and replication of mini-Chr2.

The results were different when we tested the mutants for 39-mer binding. In the presence of DnaKJ, all three mutants bound the 39-mer fragment as well as the WT RctB (Fig. 2A). In the absence of DnaKJ, binding was reduced in all cases but the reduction was larger for the WT and mutant III than for mutants IV and V. Damaging the K-I site thus appears to have different consequences for 12-mer and 39-mer binding; the former becomes defective, and the latter becomes largely independent of DnaKJ.

We then tested the DNA binding of the mutants *in vivo* using a promoter repression assay (19). There are two naturally occurring promoters in the origin of Chr2, P*rctA* and P*rctB*, both of which are repressed by WT RctB (Fig. 2A top). This is believed to be due to RctB binding to a 12-mer that overlaps P*rctA* and to a truncated 39-mer that overlaps P*rctB*. Using this assay, we found that WT RctB and mutant III were proficient in repressing P*rctA*, whereas mutants IV and V were not. In contrast, mutants IV and V were even more proficient in repressing P*rctB* than the WT (Fig. 2B). The mutants thus behaved similarly *in vivo* and *in vitro*. In the *dnaK7* host, where the *dnaK* gene is largely inactive (28), the WT and mutant III were less proficient in binding 12-mers, and mutants IV and V remained as defective as in the *dnaK*-positive (*dnaK*^+^) host. (Note that repression levels are all relative to the level of β-galactosidase values obtained with the vector alone, which is significantly less in *dnaK7* host than in the *dnaK*^+^ host). The gain in repression of P*rctB* of mutants IV and V compared to the WT was also apparent. We conclude that although DnaK is normally required to stimulate 39-mer binding, significant 39-mer binding is obtained without requiring DnaK when the K-I site of RctB is mutated. This indicates that the K-I site plays an inhibitory role in 39-mer binding (autoinhibition) that DnaK helps to overcome. The 12-mer binding, on the other hand, is not stimulated when DnaK binding to RctB is prevented either by inactivating the K-I site or by the absence of DnaK.

**FIG 3:**
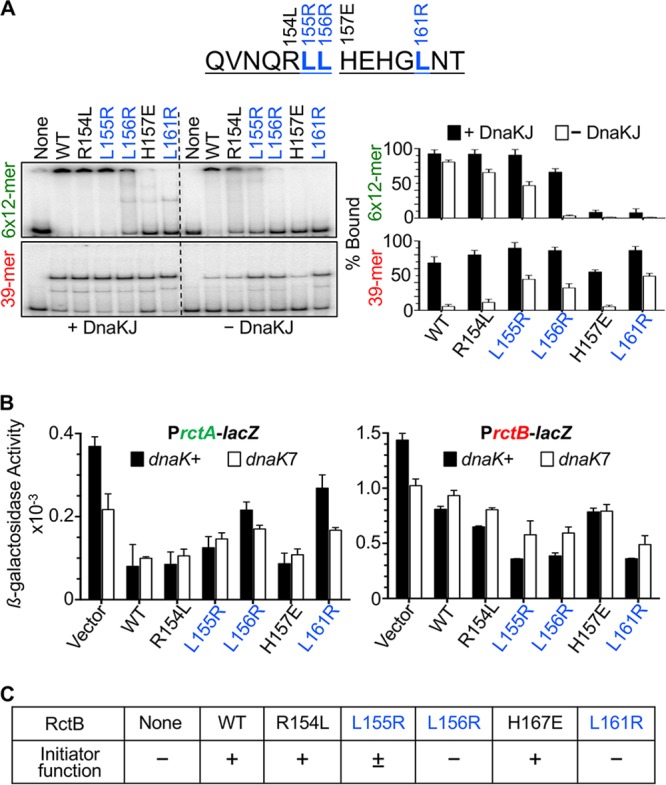
DNA binding activities of RctB mutants with single-residue changes in the DnaK interaction site (K-I site). (A) DNA binding by EMSA. The RctB mutants were R154L, L155R, L156R, H157E, and L161R. Other details are same as those described for Fig. 2A. (B) DNA binding of RctB *in vivo*. The RctB proteins were same as those described for panel A but were left untagged; otherwise, the details are same as those described for Fig. 2B. (C) Initiator activity of the RctB *in vivo*. RctB sources were same as those described for panel B; otherwise, the details are same as those described for Fig. 1E.

To examine the interaction at the residue level, we considered that DnaK prefers to recognize hydrophobic residues, particularly leucines, in client proteins (22). The hydrophobic substrate-binding cleft in DnaK has a central pocket tailored to bind leucine (23). In the stretch altered in mutants IV and V, we identified three leucines (residues 155, 156, and 161) and replaced them with arginine, a residue generally disfavored in DnaK interactions (22). We also mutated two nonleucine residues, R154 and H157. Of the five mutants, only L156R and L161R were defective in 12-mer binding both *in vitro* and *in vivo* as well as in initiator function (Fig. 3A and B). In contrast, the same two leucine mutants were more proficient than the WT in 39-mer binding *in vivo*, mimicking the phenotypes of the mutants IV and V. Of the other mutants, R154L behaved essentially as did the WT, and L155R showed an intermediate phenotype in most of the assays. The H157E mutant behaved like the WT in all the assays except for 12-mer binding *in vitro*, but this discrepancy was not investigated.

When we tested the initiator function of the mutants, we found that their activity correlated with their 12-mer binding proficiency *in vivo* (Fig. 3C). The primary inference from these results is that the change of a single leucine in the K-I site in RctB (L156R or L161R) can reduce 12-mer binding and initiator function but can increase 39-mer binding. We can explain the increased 39-mer binding *in vivo* as resulting from the combined help from the leucine mutations as well as from DnaK since the mutants were still capable of being activated by chaperones, as indicated by increased binding in the presence of DnaJK (Fig. 3A) and increased repression of P*rctB* in the *dnaK*^+^ host compared to the *dnaK7* host (Fig. 3B).

### Intragenic suppressors restore initiator function to the RctBL156R mutant.

To understand how the K-I site promotes DNA binding, we isolated mutants that suppressed the initiation defect of the L156R mutant. We mutated a plasmid carrying the L156R mutant *rctB* gene and screened a pool of mutant genes for their ability to support mini-Chr2 replication. Sequencing of 28 *rctB* genes that supported replication showed that 14 were mutated at the codon for residue M307 (changing it to isoleucine in 9, to serine in 3, and to leucine in 2) and that 12 were mutated at the codon for residue L405 (in all cases changing it to serine). In the remaining two genes, the mutation causing the L156R change reverted to the wild-type sequence.

We also mutated the plasmid carrying the L156R mutant *rctB* gene by transposon insertion *in vitro*, which resulted in insertion of five in-frame codons. In testing *rctB* genes with such insertions for initiator function, only insertions at the *rctB* codon 500 supported replication of the mini-Chr2. RctB with one such insertion was designated Tn@500 and used for further study, together with mutants M307I and L405S. We also made isogenic plasmids carrying the suppressing mutations without the mutation that caused the L156R change.

**FIG 4:**
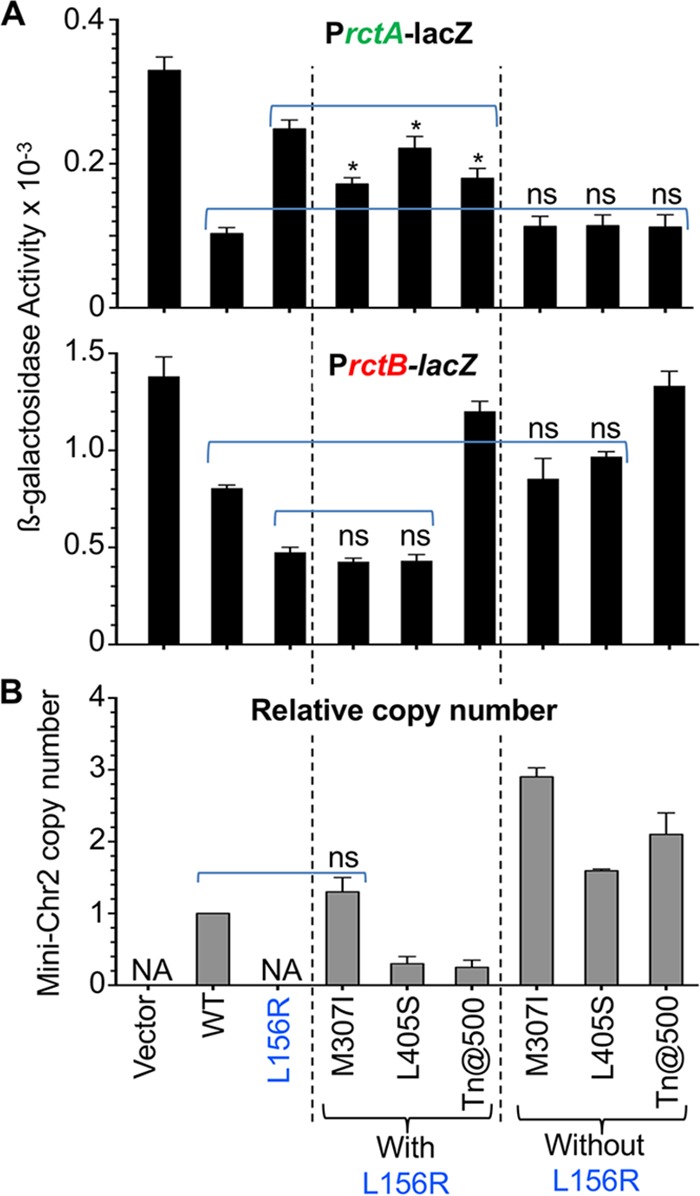
DNA binding and initiator activities of revertants of initiation-defective RctB mutant L156R. (A) DNA binding of RctB *in vivo*. The three revertants of L156R due to a second change at M307I or L405S or a transposon insertion at residue 500 (Tn@500) were tested for DNA binding as described for Fig. 2B. Binding of RctB mutants with the second changes only (without the L156R change) was also measured. The asterisks (*) indicate that in comparison to the L156R mutant results, the levels of binding in the three revertants (indicated by a bracket) were statistically distinguishable by Student’s *t* test (*P* values of 0.039, 0.025, and 0.045 for the three revertants, respectively). “ns” indicates that the binding of the mutants (indicated by a dashed line) was not significantly different from the WT results. (B) Initiator function of RctB *in vivo*. Mini-Chr2 copy numbers were determined in cells carrying different sources of RctB. “NA” indicates situations where cells did not support mini-Chr2 maintenance. The copy numbers are relative to the value obtained with WT RctB. The copy numbers are from three biological replicates.

In testing 12-mer binding, all three initiation-proficient revertants of RctBL156R showed increased binding compared to L156R alone, although the level of binding was significantly less than that of WT RctB (Fig. 4A). In contrast, without the L156R change, the mutant initiators were as proficient as WT RctB. These results suggest that the suppressing mutations alone do not affect 12-mer binding but that they help to restore the binding at least partially when this binding is defective.

The M307I and L405S changes did not significantly affect 39-mer binding with or without the L156R change (Fig. 4B). The Tn@500 mutant was disrupted in the region that we had previously determined to be important for 39-mer binding (19) and, as expected, was severely defective in 39-mer binding. The suppression thus was obtained with or without an effect on 39-mer binding.

In the *dnaK7* host, the DNA binding of the mutants was generally reduced compared to that seen with the *dnaK*^+^ host (see Fig. S3 in the supplemental material). In general, 39-mer binding was more extensively affected than the 12-mer binding, as was also evident earlier (Fig. 2), suggesting that DnaK participation is more important for 39-mer binding. The *dnaK7* host did not support replication of mini-Chr2 even when RctB was WT.

10.1128/mBio.00427-17.4 FIG S3  DNA binding of initiation-proficient revertants of initiation-defective RctB mutant L156R in *dnaK*^+^ and *dnaK7* hosts. Download FIG S3, DOCX file, 0.4 MB. Copyright © 2017 Jha et al. 2017 Jha et al. This content is distributed under the terms of the Creative Commons Attribution 4.0 International license.

To determine the initiator function of the revertants, the copy number of a mini-Chr2 plasmid was determined in *E. coli* (Fig. 4B). The copy numbers conferred by the revertants without the L156R change were significantly higher than that seen with WT RctB (the copy-up phenotype). With the L156R change, the copy numbers were reduced in all cases, indicating that the intrinsic copy-up function conferred by the suppressing mutations compensated for the defective initiator function of L156R mutant. Since without the L156R change, the 12-mer and 39-mer binding of M307I and L405S mutants was like that of the WT, their copy-up phenotype was likely due to changes in function other than DNA binding. Although 12-mer binding with the L156R change did improve to some extent in the revertants, the improvement did not correspond to the degree of the copy-up phenotype, again suggesting that changes other than in DNA binding could be involved in promoting replication (Fig. 4). A straightforward explanation of these results is that the suppressing mutations, instead of recovering 12-mer binding (a positive function), redeemed the initiation defect by reducing inhibitory (negatively regulatory) functions.

### Identification of a dimerization domain of RctB.

**FIG 5:**
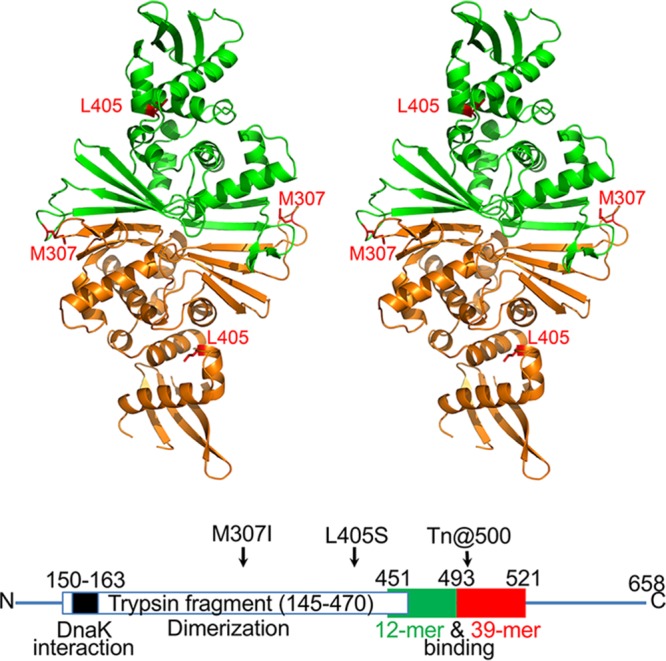
Crystal structure of the RctB fragment containing residues 145 to 470. A stereo diagram shows the tracing of the main chain of the RctB dimer, with the two protomers colored green and orange. Elements of the secondary structure are marked (see Fig. S5 in the supplemental material for more details). The side chains of the two residues (M307 and L405) where changes suppressed the initiation defect of the L156R mutant are shown in red in each protomer. The panel was prepared with PyMol (64). Also shown is a linear map of RctB marked with residue numbers of relevance to this study (not drawn to scale). The green and red boxes represent regions considered important for 12-mer and 39-mer binding, respectively (19).

In plasmids, iteron binding and plasmid replication improve by initiator mutations that reduce dimerization (29–33). Reduction in dimerization by the DnaK chaperone system is also crucial for promoting replication of these plasmids. These results prompted us to consider whether RctB dimerization plays an inhibitory role which is reduced in the suppressors. We showed earlier that a fragment consisting of the C-terminal 208 residues (ΔN450) sediments as a mixture of monomer and dimer (19). A RctB145–470 tryptic fragment that included the K-I site and the residues changed in the revertants, M307 and L405 (Fig. 5, bottom), also sedimented as a dimer (see Fig. S4A in the supplemental material). This result indicated that there could be an independent dimerization domain outside ΔN450.

10.1128/mBio.00427-17.5 FIG S4  Dimerization and DNA binding activities of an RctB fragment with residues 145 to 470. Download FIG S4, DOCX file, 0.6 MB. Copyright © 2017 Jha et al. 2017 Jha et al. This content is distributed under the terms of the Creative Commons Attribution 4.0 International license.

**FIG 6:**
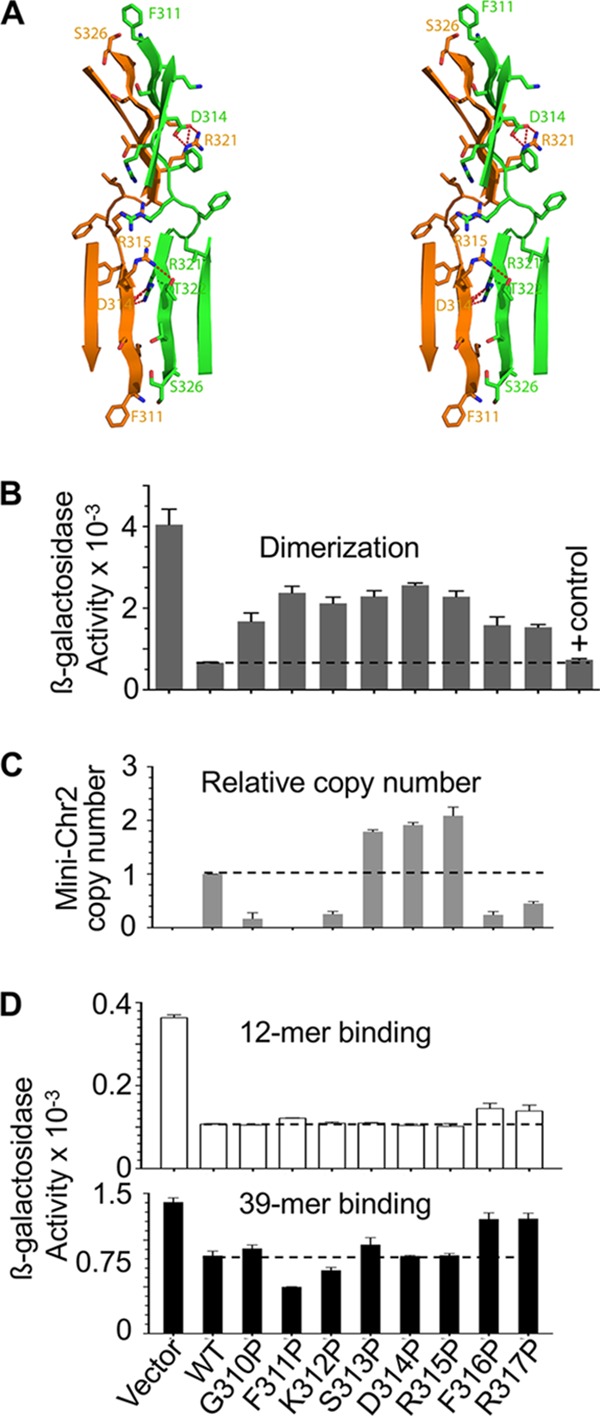
Properties of RctB mutants with residues at the dimerization interface changed to proline. (A) A stereo diagram of the dimeric interface of RctB. The side chains of selected residues are shown as sticks, with oxygen atoms colored red and nitrogen atoms blue. Residues 311 to 317 and 320 to 326 from the two monomers interact directly, forming a 7-stranded intermolecular β sheet (only the inner four strands are shown). Intermolecular hydrogen bonds between residue side chains are shown as dashed red lines. Residues 310 to 317 have been changed to proline for further studies. (B) Dimerization activity *in vivo* of proline mutants fused to λcI_N_. The dashed line is provided as a visual aid for comparison of the activities with respect to the WT RctB. (C and D) Initiator function (C) and DNA binding (D) of the proline mutants of RctB. Details of DNA binding and initiator functions are similar to those described in Fig. 4.

Further support for the N-terminal dimerization activity came from a medium-resolution crystal structure of the tryptic fragment (RctB145–470) (Fig. 5). It consists of two domains, each belonging to the α/β fold family. The traced part of the N-terminal domain contains residues 175 to 367, whereas residues 367 to 467 form the C-terminal domain. The N-terminal domain consists of five α helices, α1 to α5, sitting on top of two β sheets on both sides of strands β5 and β6, with β1, β2, β4, and β5 on one side and β3, β9, and β6 on the other side (see Fig. S5 and S6 in the supplemental material). The C-terminal domain contains three helices, α6 to α8, as well as a four-stranded β sheet, consisting of β10, β13, β12, and β11. The three helices face the helical portion of the N-terminal domain. The two molecules forming the asymmetric unit create an extended dimer through extensive interactions involving primarily the outside β5 and β6 strands of the β sheets (Fig. 6A), as well as loops 216 to 218.

10.1128/mBio.00427-17.6 FIG S5  Amino acid sequence and the secondary structure diagram of the tryptic fragment of RctB. Download FIG S5, DOCX file, 0.2 MB. Copyright © 2017 Jha et al. 2017 Jha et al. This content is distributed under the terms of the Creative Commons Attribution 4.0 International license.

10.1128/mBio.00427-17.7 FIG S6  Superposition of the coordinates of RctB and RepE. Download FIG S6, DOCX file, 4.6 MB. Copyright © 2017 Jha et al. 2017 Jha et al. This content is distributed under the terms of the Creative Commons Attribution 4.0 International license.

The independently determined crystal structure of a similar fragment of RctB (residues 155 to 483) (PDB ID 5UBD; [20]), is generally very similar to the structure described here. Most of the disordered regions missing in both sets of coordinates are the same. Although superposition of the two dimers yielded a rather large root mean square deviation (RMSD) of 1.88 Å for 476 Cα pairs, this was principally due to different rotations between the N-terminal and C-terminal domains in the two structures, most likely caused by different crystal contacts. Superposition of the N-terminal domains (residues 184 to 373) yielded an RMSD of 0.88 Å for 172 Cα pairs, not unusual for medium-resolution crystal structures. The agreement was significantly worse for the C-terminal domains (residues 374 to 467), with an RMSD of 1.4 Å for 79 Cα pairs. The larger deviation was partially due to an out-of-register segment of residues 450 to 467, incorrectly shifted by one residue in PDB ID 5UBD. However, these structural differences do not pose any significant problems with basing the interpretation of other data on either set of coordinates.

Each protomer contained two winged-helix (WH) motifs, one in the N-terminal domain and the other in the C-terminal domain. Each WH motif was similar to the prototypical WH domain of HNF-3γ (PDB ID 1VTN) (34), with an RMSD of 2.56 Å for 62 Cα atoms in the N-terminal domain of RctB and an RMSD of 2.65 Å for 49 Cα atoms of the C-terminal domain. The overall fold of the RctB fragment resembled the fold of the RepE initiator of iteron plasmid F (see Fig. S6 in the supplemental material). Whereas the two initiators were of different sizes (658 versus 251 amino acids), the tryptic fragment of RctB (325 residues) appeared to be a structural homolog of the entire RepE protein. Structures of both the monomeric form of RepE (PDB ID 1REP) (35) and its dimeric form (PDB ID 2Z9O) (8) are available complexed with DNA. In monomers of RepE and in another initiator, π protein of plasmid R6K, both the WH domains contact an iteron (36). This appears to be generally true of other iteron-plasmid initiators, as determined by modeling (37, 38). A conformational change in the N-terminal WH domain makes the plasmid initiators bind as dimers through their C-terminal WH domains to inverted repeats of iteron half-sites (7, 8). The residue changed in one of the revertants (M307) was in a loop connecting the two β-strands of the dimerization interface, and the residue changed in the other (L405) was in the turn of the HTH motif of the C-terminal WH domain (Fig. 5). Following the plasmid paradigm, the positions changed in the revertants indicate that the middle region of RctB is involved both in dimerization and in DNA binding. A similar inference has been drawn independently (20).

As shown by sedimentation analysis, dimerization of RctB145-470 was reduced in the presence of the L156R+M307I substitutions but not in the presence of the L156R+L405S substitutions (see Fig. S4A in the supplemental material). The dimerization defect of the M307I change was also evident in a λcI-based dimerization assay *in vivo* (see Fig. S4B in the supplemental material). The assay seems to have been more sensitive, as it revealed that L156R was also dimerization defective. The tryptic fragment failed to bind to a DNA probe with 6×12-mers (Fig. S4C). The WH motifs thus appear insufficient for 12-mer binding, which is consistent with our previous finding that residues at further C-terminal locations (up to position 493) are important (Fig. 5, bottom) (19). The inference is also consistent with the finding that RctB can be split without losing its initiator function (see Fig. S7A in the supplemental material). Splitting at both residues 417 and 450, which should be interrupting the C-terminal WH motif, still allowed initiator function, whereas splitting at residue 491 did not, consistent with the latter interrupting the region considered important for 12-mer binding.

10.1128/mBio.00427-17.8 FIG S7  Interactions of the N- and C-terminal fragments of RctB. Download FIG S7, DOCX file, 0.5 MB. Copyright © 2017 Jha et al. 2017 Jha et al. This content is distributed under the terms of the Creative Commons Attribution 4.0 International license.

The failure of the tryptic fragment of RctB to bind 6×12-mers could also result from structural differences between RctB and RepE. Each protein includes two clearly identifiable domains, the relative orientations of which are quite variable, as seen in their crystal structures (see Fig. S6A in the supplemental material). Superposition of the RepE dimer (PDB ID 2Z0O) on RctB yielded an RMSD of 3.8 Å for 225 Cα pairs that belong to the N-terminal domains only, leaving the C-terminal domains far from each other. Nevertheless, superposition of the C-terminal domain of RepE (residues 160 to 247) on the C-terminal domain of RctB yielded an RMSD of 3.6 Å for 79 Cα pairs, indicating that only the relative orientations of the domains were changed and not their structures (see Fig. S6B in the supplemental material). However, superposition of the monomeric RepE (PDB ID 1REP) on RctB yielded an RMSD of 3.3 Å for 214 Cα atoms belonging to both domains, with the relative orientations of the N- and C-terminal domains very similar in the two structures (see Fig. S6C in the supplemental material). It is surprising that the relationship between the domains of the dimeric RctB was very similar to that seen with monomeric RepE, although the dimer interfaces are quite similar for these two proteins. The fold of the N-terminal domain of pPS10 RepA (PDB ID 1HKQ) was even more similar to that of RctB, with an RMSD of 2.5 Å for 198 Cα pairs belonging to both molecules forming the dimer (not shown). Similarly to RctB, the structure of pPS10 RepA was determined in the absence of DNA. The “choice” between one arrangement and the other for the two WH domains might be determined by the length of the intervening DNA sequence between the half-repeats in each kind of dyad. A unique feature of the dimer interface area of RctB, not seen in either RepA or RepE, is the long β hairpin made of residues 327 to 341 which extends the innermost strands of the intermolecular β sheet and adds additional intermolecular contacts that stabilize the dimer (see Fig. S6A in the supplemental material).

### Interaction of the K-I site with other regions of RctB.

The crystal structure of RctB, in which the K-I site was unfolded, was not helpful in understanding how that region promotes DNA binding. To test whether the region could be interacting directly with other regions of RctB, we determined whether the N- and C-terminal fragments of RctB could interact in *trans*. The interaction was evident both *in vivo* and *in vitro* (see Fig. S7B and C in the supplemental material). The interaction was also indicated by chemical cross-linking with DTSSP [3,3′-dithiobis (sulfosuccinimidyl propionate)]. DTSSP specifically cross-links lysine residues that are within 10 Å of each other. When RctB145-470 was used, reduced crosslinker adducts were reproducibly observed on K168 and K404, suggesting a cross-link between these two residues in the dimeric protein (see Fig. S8A in the supplemental material). The two lysine residues are located in peptides 145 to 209 and 386 to 420. The first peptide includes the K-I site, and the second one includes the residue L405 that was changed in one of the suppressors. In other words, the K-I site could be directly contacting the C-terminal WH domain. With intact RctB, four new cross-links were observed in the dimer: one between K196 and K520 and the other three (K476 to K520, K495 to K520, and K520 to K520) involving the region that we previously showed to be important for dimerization and DNA binding (19). The cross-linking of K520 with itself indicates that the residue is in or within 10 Å of a dimerization interface, consistent with our earlier observation that there is a dimerization domain in the C terminus of RctB. These results suggest the possibility that the K-I site could be directly contacting the region responsible for 39-mer binding to cause autoinhibition. The reduced interaction between the C-terminal fragment and the N-terminal fragment with the L156R change is consistent with this inference (see Fig. S8B in the supplemental material). We note that the N-terminal to C-terminal interactions could also occur between two different monomers arranged in a head-to-tail orientation. This configuration would result in the formation of a DNA-bound protein filament, mimicking to some extent a structure proposed previously for RepA handcuffs (39).

10.1128/mBio.00427-17.9 FIG S8  Intra- and intermolecular interactions of RctB. Download FIG S8, DOCX file, 0.3 MB. Copyright © 2017 Jha et al. 2017 Jha et al. This content is distributed under the terms of the Creative Commons Attribution 4.0 International license.

### Replication regulatory role of the dimerization domain of RctB145–470.

To establish the significance of the dimerization as seen in the crystal, we individually changed residues 310 to 317 of the dimerization interface to proline (Fig. 6A). Proline was chosen because in RepE of F plasmid, the R118P change in the dimerization interface eliminated the dimerization activity, made iteron binding of the monomers independent of chaperones, and conferred the copy-up phenotype (40). We reasoned that if any of the RctB proline mutants could reduce dimerization and confer the copy-up phenotype, then an inhibitory role for the dimerization domain could be argued.

**FIG 7:**
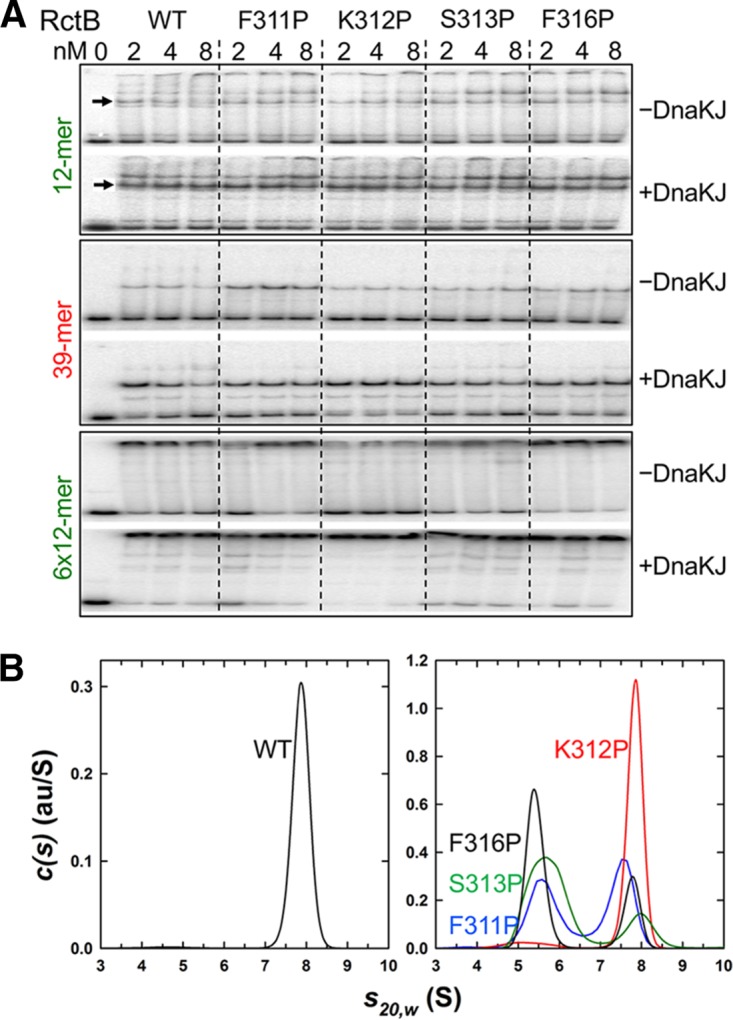
DNA binding and dimerization of representative RctB mutants with residues changed at the dimerization interface. (A) EMSA. Four mutants, F311P, K312P, S313P, and F316P, were studied for binding to a single 12-mer, a single 39-mer, and an array of 6×12-mers in the absence and presence of DnaKJ. (B) Sedimentation velocity absorbance c(*s*) profile for WT MBP-RctB at 0.9 μM based on data collected at 280 nm (left panel) and for its derivatives F311P (blue), K312P (red), S313P (green), and F316P (black) at 0.4 μM based on data collected at 230 nm (right panel). The c(*s*) profiles of WT MBP-RctB at multiple concentrations show the presence of a single dimeric species at 7.90 S having an estimated molar mass of 215 kDa. At loading concentrations of 0.4 (shown) and 1.2 μM, the c(*s*) profiles for the samples represented in the right panel indicate the presence of a reversible monomer-dimer equilibrium with the presence of both the MBP-RctB monomer and dimer. au/S, absorbance units per Svedberg.

All the proline mutants were partially defective in dimerization *in vivo* (Fig. 6B). They were also changed in initiator function: some (S313P, D314P, and R315P) showed the copy-up phenotype, whereas the rest were reduced in initiator function (Fig. 6C). The copy numbers were not correlated with binding proficiency to either 12-mer or 39-mer *in vivo* (Fig. 6D) and *in vitro* (Fig. 7A). These results indicate that the dimerization interface seen in the crystal plays a significant role in controlling mini-Chr2 replication without significantly affecting DNA binding.

We found that the DNA binding of the proline mutants increased in the presence of DnaJK *in vitro* (Fig. 7A). Importantly, the lower shifted band of the 12-mer fragment and the single shifted band of the 39-mer fragment, both representing monomer binding (17, 41), increased in the presence of chaperones. This result indicates that the active fraction of monomers increases in the presence of chaperones. At higher RctB concentrations, both the lower and upper shifted bands of 12-mer increased, indicating that dimers are generated from the chaperone-activated monomers. The four proline mutants (F311P, S313P, F316P, and K312P), representing different initiator and DNA binding properties, were all reduced in dimerization to different degrees (Fig. 7B). In sum, these results are consistent with a role of the chaperones in activating monomer binding irrespective of the dimerization status of the initiator.

## DISCUSSION

The Chr2 replicon is organized similarly to the replicons of iteron plasmids, but their characteristics with respect to the timing of replication initiation in the cell cycle differ: the former initiates at a specific time of the cell cycle, the norm in chromosomal replication, whereas the latter initiate at no fixed time in different cells of a growing culture (16). A comparative study of the two replicon types may thus reveal the underlying mechanisms that changed the random timing of replication initiation to fixed timing in the cell cycle.

In plasmid-to-chromosome transitions, Chr2 seems to have retained all the control features of iteron plasmids but has added quite a few new ones (16). The central feature of control remains the initiator-origin interactions. In plasmids, initiator binding to iterons serves to promote as well as inhibit replication. The latter becomes the overriding feature when plasmid copy number increases. In Chr2, the role of 12-mers, the iteron analogs, is restricted to initiation, and a second kind of site, 39-mer, was acquired to inhibit replication. Chr2 has retained the plasmid feature of dependence on the DnaK chaperone system for initiator binding not only to 12-mers but also to 39-mers. Here we show that the chaperones promote the two binding activities through distinct mechanisms.

### Differing roles of DnaK in the control of replication-promoting 12-mer and replication-inhibiting 39-mer binding.

In plasmids, the chaperones remodel the dimerization domain of the initiator, which results in reduced dimerization and increased monomer binding and initiator function (5, 7, 8). Three lines of evidence suggest that a similar mechanism operates on the Chr2 initiator RctB. (i) RctB has a dimerization domain that is folded similarly to the dimerization domains of plasmid initiators (Fig. 5) (20). (ii) Chaperones increase RctB monomer binding (Fig. 7A; see Fig. S4D in the supplemental material). (iii) Reduction in dimerization by mutation does not bypass the chaperone requirement for DNA binding (Fig. 7A). These observations suggest a requirement for remodeling of the dimerization domain to promote 12-mer binding and initiator function.

RctB binding to 39-mers, however, may not require remodeling. Inactivation of the K-I site on RctB that abrogates 12-mer binding renders 39-mer binding independent of DnaK. This indicates that the K-I site is inhibitory for 39-mer binding, and we believe that DnaK binding to the site shields it from exerting its inhibitory effect.

**FIG 8:**
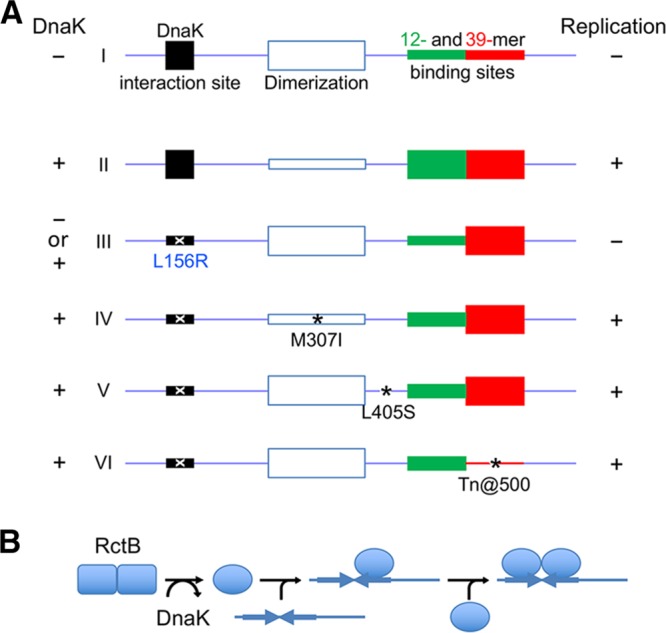
(A) A pictorial summary of activation of DNA binding of RctB by DnaK. Functional regions of RctB are represented as rectangles on a linear map of the initiator. Thinner rectangles indicate reduced activities. (I) In the absence of DnaK, both the 12-mer and 39-mer binding sites remain inactive but for different reasons: the former site cannot fold properly without interaction with DnaK, and the latter is inhibited by the DnaK interaction site (K-I site), possibly by direct contacts (autoinhibition). The improperly folded 12-mer region cannot support replication. (II) The autoinhibition is released by shielding of the K-I site by DnaK binding. DnaK interaction remodels RctB, which reduces dimerization and activates the monomers for DNA binding. (Note, however, that reduction in dimerization by mutating the dimerization domain does not obviate remodeling of monomers [not shown]). (III) The autoinhibition is also released by changes such as that represented by L156R in the K-I site. The mutation prevents DnaK interaction, and, without DnaK participation, the 12-mer region remains inactive. (IV to VI) The initiation defect due to the L156R change can be suppressed by second-site changes (*) that partially activate the 12-mer binding region but primarily reduce negative regulatory activities such as dimerization (IV), an uncharacterized activity from a WH domain (V), and 39-mer binding (VI). Full 12-mer activity, as well as a reduction in one of the negative activities, causes the copy-up phenotype (not shown). (B) Sequential binding of remodeled RctB monomer to a 12-mer. DnaK remodels RctB into a form that reduces its dimerization. The monomers bind to one half of an inverted repeat present in 12-mer sites. Upon an increase of remodeled RctB levels, both half-sites are occupied either by binding of a second monomer or binding of a dimer formed in solution (not shown). The details of protein-protein and DNA-protein interactions remain speculative.

The same leucine residues in the K-I site of RctB that are required to activate 12-mer binding by DnaK are also required to inhibit 39-mer binding. This suggests that DnaK binding to the same site on RctB plays different roles in the two binding mechanisms. In the one case, the binding leads to remodeling, and in the other, mere binding appears to suffice. The absence of a remodeling requirement is distinct from the mechanisms by which the DnaK system promotes replication in bacteriophage lambda, iteron plasmids, and *C. crescentus*. The lack of 39-mers in iteron plasmids suggests that the Chr2 plasmid progenitor acquired extra residues (residues 493 to 521) for binding to 39-mers, along with a distinctive mechanism to activate the binding by chaperones. Our current understanding of how DnaK contributes to DNA binding in Chr2 is summarized in Fig. 8.

### Multiple modes of control of Chr2 replication.

Studies in *E. coli* have established that replication initiation is regulated by a combination of positive and negative regulatory factors (42). The initiation defect due to a deficiency in a positive regulator can be compensated by reducing the efficacy of a negative regulator. The defective 12-mer binding and initiator function of the RctB L156R mutant are redeemed by any of three intragenic mutations without approaching the wild-type level of 12-mer binding. This suggests that the initiation defect was suppressed primarily by reducing negative regulatory mechanisms. The locations of the suppressing changes were distinct: one (M307I) was in the dimerization domain, one (L405S) was in a winged-helix (WH) domain, and one (Tn@500) was in a region important in 39-mer binding. These three changes, and only these changes, were repeatedly found, suggesting that they are affecting key negative regulatory functions of RctB, as we discuss below.

Dimerization of RctB was reduced by the substitution M307I (see Fig. S4 in the supplemental material). Because the mutant showed the copy-up phenotype (Fig. 4), the reduction in dimerization likely reduced a negative regulatory action, such as handcuffing. Other changes in the dimerization interface also showed the copy-up phenotype with an insignificant change in 12-mer binding (Fig. 6 and 7).

The L405S change did not reduce dimerization (see Fig. S4 in the supplemental material). It occurs within a WH domain, a conserved feature in replication initiators (43), and is one of three such domains that contribute to DNA binding (20). The domain could be split without losing the initiator function (see Fig. S7 in the supplemental material). The L405S change is next to the K404 residue that interacts with the K-I site and thus might partially fulfill the role of the K-I site (see Fig. S8A in the supplemental material). How the L405S change confers the copy-up phenotype remains to be understood.

The copy-up phenotype of the Tn@500 mutant is easily explained because the 39-mer binding region, which serves an inhibitory function, was interrupted in this mutant (17, 41). The reverse is also true: in the F311P mutant, 39-mer binding increased both *in vivo* and *in vitro*, and replication was inhibited (Fig. 6C and D; Fig. 7A). The copy-up phenotype of the Tn@500 mutant is almost surely a result of reduced 39-mer binding.

### Paradoxical findings.

Chaperones promote both 12-mer binding and 39-mer binding, which have opposite effects on replication. Although seemingly paradoxical, this could be suggesting the importance of controlling both positive and negative regulatory steps to achieve stringent replication control. The chaperones also promote binding of both monomers and dimers to 12-mers (Fig. 7A). The increase of dimer binding is the very antithesis of what the chaperones do to iteron binding. The M307I change and some of the other changes in the dimerization interface, although effective in reducing dimerization, did not reduce dimer binding to 12-mers. How can the reduction in dimerization promote dimer binding? One possibility is that only monomers bind to 12-mers and the apparent dimer binding results from DNA-mediated association of two monomers, as has been postulated for other DNA binding proteins (Fig. 8B) (44, 45). The active monomers can also dimerize in solution to form “active” dimers before DNA binding. The dimer binding is consistent with the fact that 12-mers but not iterons have an inverted repeat, TGATCA, which is essential for DNA binding (46). In principle, two monomers can each contact one half of the inverted repeat, as is the norm in many dimeric DNA binding proteins. However, monomer binding to a site with an inverted repeat is unprecedented.

### Plasmid-to-Chr2 transition.

The introduction of the dimer binding capacity required a major change to iterons: inclusion of an asymmetrically placed inverted repeat, which also allows the sites to be methylated by the Dam methylase and to be controlled by the hemimethylated DNA binding protein SeqA (47). Another major change from the iteron plasmids is the delegation of the inhibitory role of the iteron to 39-mers. Biological processes that are controlled by separate positive and negative effectors (dual inputs) offer a more stringent and robust form of control because they can be independently modulated. The 39-mers do not contain methylation sites and bind monomers only, allowing them to be controlled independently. In plasmids, when the copy number increases so does the initiator concentration. This creates more dimers and increases their inhibitory action (e.g., by handcuffing). By the same token, the postinitiation increase of dimers can promote dimer binding to 12-mers, arguably an inhibitory function. The dimer binding option may thus have afforded additional negative regulatory capability. In that case, the chaperones could be contributing to negative regulation by promoting dimer binding to 12-mers as well as monomer binding to 39-mers. Thus, the chaperones might be remodeling the initiators as in the case of iteron plasmids; the binding results that follow have different impacts on DNA binding in Chr2. In plasmids, reduction of initiator dimerization increases copy number as well as iteron binding. In Chr2, initiation can increase without changing 12-mer binding.

The importance of the dimerization domain in Chr2 replication control is evident from previously isolated RctB mutants, which were found to confer the copy-up phenotype or to allow initiation when the WT protein could not. The majority of these mutants have changes in the region containing the dimerization domain (145 to 470) (see Fig. S8A in the supplemental material). RctB is also remodeled by binding to a novel site, *crtS*, that activates the initiator and helps it to function in the absence of DnaK (46, 48). Residue changes that bypass *crtS* or DnaK dependence are found in nearby regions of RctB (red or green changes), suggesting relatedness in the functioning of the two remodelers. Additional structural studies of RctB are in order to understand further the different forms of the initiator and their role in replication control.

## MATERIALS AND METHODS

### Strains and plasmids.

These are listed in Table S1 in the Text S1 file in the supplemental material.

10.1128/mBio.00427-17.1 TEXT S1  Methods and Tables S1 to S3. Download TEXT S1, DOCX file, 0.1 MB. Copyright © 2017 Jha et al. 2017 Jha et al. This content is distributed under the terms of the Creative Commons Attribution 4.0 International license.

### Mutagenesis.

Mutations creating alanine substitutions in the K-I site of RctB were generated by the PCR method of overlap extension using a Kapa HiFi HotStart ReadyMix PCR kit (Kapa Biosystems) (49). N16961 DNA was used as the template. The primers are described in Table S2 in the Text S1 file. The PCR products were cloned either in pET22b vector between NdeI and XhoI sites for promoter repression assays or in pMAL-c2X vector between EcoRI and SalI sites for DNA binding and pulldown assays *in vitro*. Alanine mutants I to VI were generated by this procedure. The mutations were verified by DNA sequencing in all cases.

N-terminal deletion mutants of RctB were generated by PCR, and the products were cloned in pET22b and pMAL-c2X vectors as described above. The point mutants of RctB were generated by site-directed mutagenesis using a Kapa Biosystems PCR kit as described above.

Mutations suppressing the replication defect of the L156R mutant *rctB* gene were isolated in a plasmid carrying the mutant gene (pJJ263). The *in vivo* mutagenesis was done by transferring the plasmid into the mutator strain, *E. coli* XL-1 Red (Agilent Technologies), and growing the transformants on plates under conditions of selection for 36 h at 37°C. The mutagenized pool of plasmids was used to transform DH5α cells, and the transformants that supported mini-Chr2 replication were selected.

The pJJ263 plasmid was also mutagenized *in vitro* by transposon insertions using a mutation generation system (Thermo Scientific/Finnzymes). This system inserts five in-frame codons (50). Four separate insertion reactions were performed, each in a total volume of 20 µl containing 100 ng of Entranceposon (M1-chloramphenicol resistance [M1-Cm^r^]) as the donor DNA and 300 ng of pJJ263 as the target DNA. Following incubation at 30°C for 1 h, reaction products were used to transform DH5α cells. The transposon-containing plasmid clones were selected on LB-ampicillin-chloramphenicol (LB-Ap-Cm) plates. A total of ~1 × 10^4^ colonies were pooled, and their plasmid DNA was isolated and digested with NdeI and XhoI with sites at the ends of *rctB*. The 3.1-kb fragment carrying the transposon-inserted *rctB* gene was gel purified and ligated to NdeI-XhoI-digested pET22b. The ligation mixture was used to transform DH5α, and insert-containing plasmid clones were selected on LB-Ap-Cm plates. A total of ~1 × 10^4^ colonies were pooled and grown in LB-Ap-Cm liquid medium at 37°C for 2 h, after which plasmid DNA of the culture was isolated. The transposon was eliminated from the plasmids by digestion with NotI. The linear plasmid backbone was gel purified, recircularized, and used to transform DH5α, and the transformants were selected on LB-Ap plates. Plasmid DNA isolated from pooled transformants comprised the final insertion mutant library. The library was used to transform DH5α cells, and the transformants that supported mini-Chr2 replication were selected. The randomness of insertion was tested by PCR using a primer matching an end of *rctB* and another (NotI) primer that is internal to the 5-codon insertion. This yielded a smear of products ranging from ~2 kb to ~100 bp, consistent with insertions being distributed across *rctB*.

The pJJ114 mini-Chr2 plasmid was made by digesting pTVC31 with PstI and cloning the *cat* gene with PstI ends from pSP102 (51).

### Protein purification and partial proteolysis.

WT and mutant RctB proteins were purified using pMal-c2X or pET22b or pET28a vector and BL21(DE3) as the host. MBP-tagged proteins were purified as previously described (17). His-tagged proteins were purified after induction with 0.4 mM IPTG (isopropyl-β-d-thiogalactopyranoside) at an optical density at 600 nm (OD_600_) of approximately 0.6 at 37°C, and growth was continued overnight at 16°C. The cells were lysed in a buffer containing 50 mM sodium phosphate (pH 8.0), 500 mM NaCl, 20 mM imidazole, 1 mM EDTA, 10% glycerol, and 3 mM dithiothreitol (DTT). The cells were lysed by sonication with 5-s-on/10-s-off cycles for 10 min. The lysates were subjected to centrifugation for 30 min at 40,000 × *g*. Cleared lysates were passed through 0.45-µm-pore-size filter paper (Millipore) and then passed through the Ni column preequilibrated with the buffer described above. The flowthrough was passed through the same column once more, followed by a 20-column-volume wash with the same buffer. The recombinant proteins were eluted with the same buffer containing 200 mM imidazole. The eluted proteins were immediately passed through a PD10 desalting column and next through a Superdex 200 column (GE Healthcare) equilibrated with a buffer containing 20 mM Tris-HCl (pH 8.0), 300 mM NaCl, 5% glycerol, 1 mM DTT, and 1 mM EDTA. The proteins were dialyzed overnight at 4°C against the same buffer, and their concentrations were determined using a NanoVue spectrophotometer (GE Healthcare) and by SDS-PAGE, using bovine serum albumin (BSA) as the standard. RctB145-470 L156R used for crystallography was His tagged, and the concentration was adjusted to 25 mg/ml using Amicon 10 K filters (Millipore).

Partial proteolysis of RctB was performed using trypsin. A few relatively stable RctB fragments, as judged by SDS-PAGE, were analyzed by mass spectrometry (MS), and they spanned residues 145 to 420, 145 to 463, 145 to 470, 155 to 372, and 155 to 385. The largest fragment, spanning residues 145 to 470, was chosen for further studies.

### Co-IP and Western blotting.

DnaK interaction with RctB was performed in buffer A (20 mM Tris-HCl [pH 7.4], 100 mM potassium glutamate, 0.1 mM EDTA, 1 mM DTT, 0.1% IGEPAL CA-630, 5% glycerol) containing 1 µM RctB, 1 µM DnaJ, 2 µM DnaK, and 100 µM ATP in a 50-µl reaction mixture. The mixtures were incubated for 30 min at 4°C with rocking and diluted 10-fold with buffer B (50 mM Tris-HCl [pH 7.4], 100 mM NaCl, 0.1% Tween 20). Subsequently, a 20-µl slurry of amylose magnetic beads (NEB no. E8035) in buffer B was added, and the reaction mixture was incubated for 2 h at 4°C with rocking. The magnetic beads were collected and washed 4 times with buffer C (50 mM Tris-HCl [pH 7.4], 300 mM NaCl, 0.2% Tween 20, 0.1 mM EDTA). The bead-bound proteins were eluted with 1× SDS PAGE loading buffer, analyzed by SDS-PAGE, and transferred to nitrocellulose membranes. Following the transfer, RctB was detected with RctB antibody (17) and DnaK with DnaK antibody (Stressgen Biotechnologies). In some cases, cell extracts taken at an OD_600_ of 0.3 were used instead of purified RctB (Fig. 1B).

### Y2H assay.

RctB and DnaK interaction was studied using a Brent yeast two-hybrid system (52). EGY48 yeast cells [his3 trp1 ura3 LexAop (x6)-LEU2] were grown and transformed with the following vectors: (i) pEG202, encoding the DNA binding domain of LexA and a *HIS3* selectable marker; (ii) pJG4-5, encoding the activation domain of B42 and a *TRP1* selectable marker; and (iii) pSH18-34, containing the *lacZ* gene under the control of the *lexA* operator and a *URA3* selectable marker (53). The genes were amplified using a PCR kit (Kapa Biosystems). N16961 DNA was used as the template, except for the alanine mutants, where pJJ242-244 plasmids were used. The PCR products of the genes for WT RctB and the ΔN100 and ΔN200 mutants were cloned in pEG202 between EcoRI and XhoI sites, which resulted in plasmids pJJ218, pJJ224, and pJJ225, respectively. The PCR products of alanine mutants III, IV, and V were also cloned in pEG202, which resulted in plasmids pJJ253, pJJ254, and pJJ255, respectively. The PCR product of the gene for DnaK was cloned at the pJG4-5 vector at an XhoI site, which resulted in plasmid pJJ223.

The transformants were selected on glucose plates lacking histidine, uracil, and tryptophan, purified, and grown in medium lacking histidine, uracil, and tryptophan but containing 2% galactose and 1% raffinose. The cells were spotted after serial dilution on plates lacking histidine, uracil, tryptophan, and leucine but containing galactose and raffinose.

### Dimerization of RctB fused to λ repressor.

RctB dimerization was tested *in vivo* after fusion to the N-terminal domain of λ repressor (λcI_N_) (6). We used either pJJ112 (P*lac*-λcI_N_-WT*rctB*) or pJJ417 (P*lac*-λcI_N_-*rctB*L156R) plasmid as the template. Mutations causing the M307I and L405S changes, and in residues 310 to 317 at the dimerization interface to proline, were introduced by site-directed mutagenesis. The assay was most discriminatory when uninduced levels of fusion proteins were tested.

### Electrophoretic mobility shift assay (EMSA).

Plasmids (5 µg) were digested with appropriate restriction enzymes followed by dephosphorylation of the ends with shrimp alkaline phosphatase (SAP) (Promega). The desired fragments were gel purified and radiolabeled with 30 units of polynucleotide kinase (NEB) and 50 µCi of adenosine 5′-[γ-^32^P]triphosphate (PerkinElmer). The labeled fragment was purified through G-50 columns (Roche Diagnostics Corporation). Further details were as described previously (17).

### β-Galactosidase assay.

Monolysogen of λ phage carrying promoter fusion P*rctA-lacZ* (λDKC383) or P*rctB-lacZ* (λDKC382) was used. The phages were obtained by UV induction of lysogens CVC1798 and CVC1797, respectively (19), and used to lysogenze *recA* hosts BR8706 (*dnaK*^*+*^) and BR4390 (*dnaK7*) (19, 54). The *dnaK7* strain was used at the permissive temperature of 30°C (28). The lysogens were followed on indicator plates and transformed with desired plasmids, and the β-galactosidase activities of the transformants were determined (17).

### Initiator activity *in vivo*.

RctB was considered proficient in initiation when it could support replication of a mini-Chr2 plasmid under conditions of selection. The activity was measured quantitatively by monitoring mini-Chr2 copy numbers. DH5α cells were transformed with a RctB source plasmid, and the transformants were further transformed with a mini-Chr2 (pJJ114) plasmid. Where the second transformation was successful, plasmid DNA was isolated from cells obtained at an OD_600_ of 4 and added to a fixed volume of the DH5α/pNEB193 cells (recovery control). For each RctB source, the mini-Chr2 band intensity was normalized first with respect to the intensity of the pNEB193 band and then with respect to the intensity of the mini-Chr2 band when the source of RctB was WT (11).

### Sedimentation velocity.

The data were collected at 50,000 rpm and 20°C using an absorbance optical detection system at 230 or 280 nm and an Optima XL-A analytical ultracentrifuge (55). The samples were dissolved in a mixture containing 20 mM Tris (pH 8.0), 300 mM NaCl, 1 mM TCEP [tris(2-chloroethyl) phosphate], 1 mM EDTA, and 5 mM MgCl_2_ (Fig. S4A in the supplemental material) or in the same buffer where MgCl_2_, was replaced with 40 mM maltose (to avoid aggregation) (Fig. 7B) and were analyzed at multiple concentrations. Data were analyzed as previously described (19).

### Protein crystallization and structure determination.

Crystals of the L156R mutant of the tryptic fragment of RctB (residues 145 to 470) in which methionine was replaced by seleno-l-methionine (SeMet) were grown using the hanging-drop method. The well solution contained 1.5 M ammonium sulfate and 0.1 M sodium acetate buffer at pH 4.5. The protein was concentrated to 21 mg/ml in a mixture containing 20 mM Tris-HCl (pH 8.0), 0.3 M NaCl, 1 mM DTT, 1 mM EDTA, and 5% glycerol. The hanging drop was a mixture of 4 µl of sample and 2 µl of well solution. Crystals grew in 2 to 7 days, reaching the size of ~0.1 by 0.1 by 0.05 mm. Diffraction data extending to 3.0 Å were collected at 100 K at beamline ID-22 (SER-CAT) at the Advanced Photon Source, Argonne National Laboratory. Diffraction frames were processed with Denzo and Scalepack (56). The structure was determined by single-wavelength anomalous diffraction (SAD). Fourteen of the expected 20 Se atoms (not counting the initiator methionine that was likely to be disordered) were located with the program SHELXD (57), with one correct phase trial of 152 resulting in CC all/weak of 37.5/21.0 (all other trials had less than 18.6/6.0). The initial set of phases was improved by solvent flattening, density modification, and noncrystallographic symmetry averaging using several modules of HKL3000 (58). The initial model was built with Buccaneer (59), and the resulting map was improved by one round of Rosetta refinement (60). The missing loops and the C-terminal 40 residues were built manually with COOT (61), and the structure was refined with Phenix (62). The asymmetric unit contains a 2-fold symmetric dimer of RctB. The final model does not include residues 145 to 174, 242 to 255, and 468 to 470 of either protomer, since the respective electron densities were not visible. Solvent was not modeled due to the relatively low resolution of the data. Data collection and refinement statistics are shown in Table S3 in the Text S1 file.

### Accession number(s).

The coordinates and structure factors determined in this work have been deposited in the Protein Data Bank (PDB ID 5TBF).
