# Supplementary material for: The DnaK Chaperone Uses Different Mechanisms To Promote and Inhibit Replication of Vibrio cholerae Chromosome 2
Source: mBio. 2017 Apr 18;8(2):e00427-17. doi: 10.1128/mBio.00427-17 (PMC5395669; doi:10.1128/mBio.00427-17)
Supplement: FIG S1 [file mbo002173276sf1.docx]

**
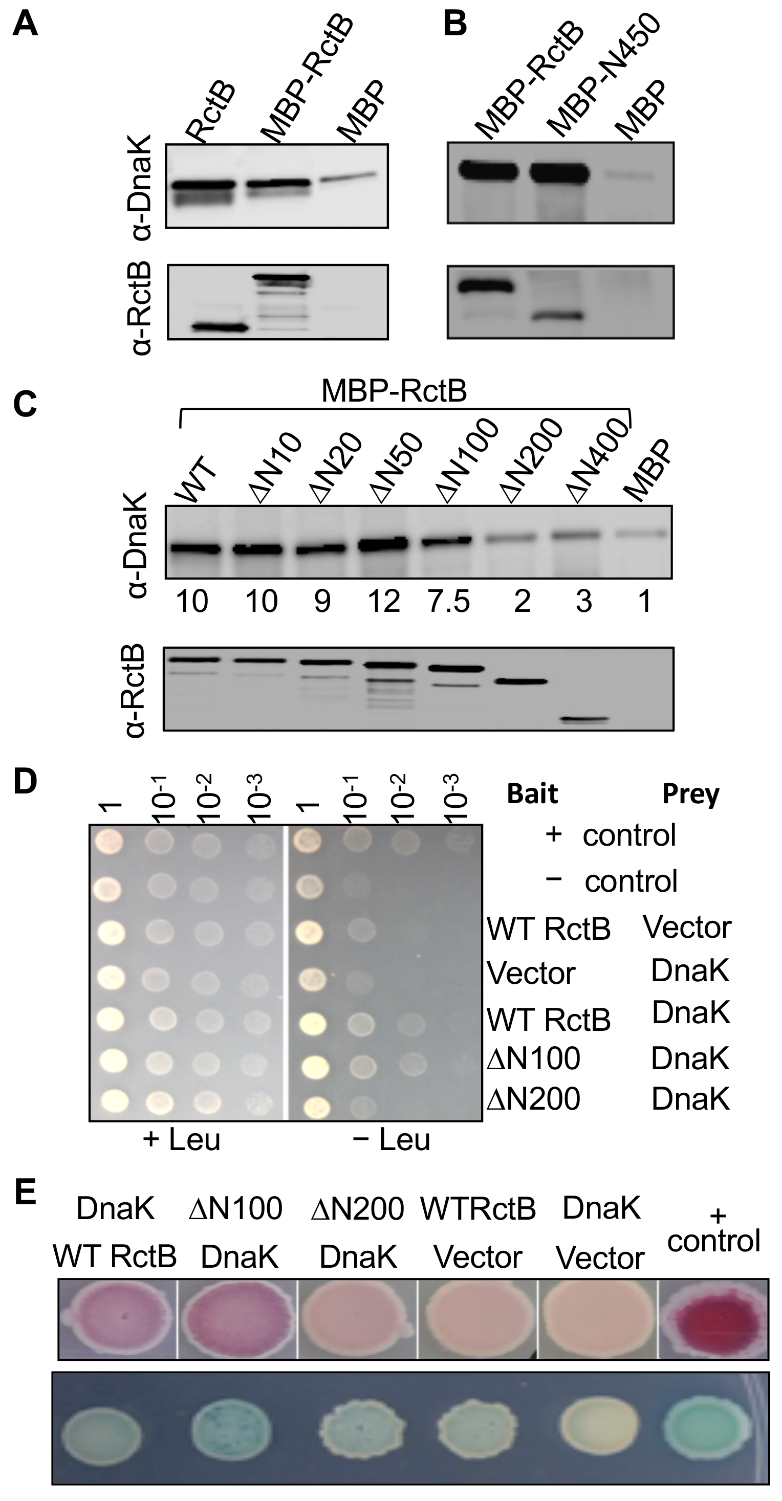
Fig. S1. Identification of an RctB region important for interaction with DnaK *in vitro* and *in vivo*. (A-C)** Interaction of RctB with DnaK by co-immunoprecipitation. (A) RctB WT without or with an N-terminal MBP tag was incubated with DnaKJ and ATP, and then treated with rabbit anti-RctB antibody (α‑RctB) and IgG magnetic beads to pull down untagged WT RctB. In all other cases the pull down was with amylose magnetic beads. The beads upon pull‑down were subjected to SDS-PAGE followed by Western blot analysis with α‑RctB and antiserum against DnaK (α‑DnaK). The MBP tag alone was used as a negative control. (B) Same as (A) except that a deletion mutant carrying the N-terminal 450 residues of RctB (N450) was also used. (C) Same as (B) except that new N-terminal deletion derivatives were used. (D) Interaction between RctB and DnaK by the yeast two hybrid (Y2H) assay. Plasmids pSH17-34 and pRFHM1 were used as positive and negative controls, respectively. RctB WT and its two N‑terminal deletion derivatives (∆N100 and ∆N200) were cloned in the Y2H bait vector (pEG202) and DnaK was cloned in the Y2H pray vector (pJG4-5). The yeast cell EYG48 was transformed with the above clones individually and the transformants were spotted on indicator plates with and without Leu after serial dilutions as identified in the top of the figure. The bait and prey fusions of RctB and DnaK were also tested with corresponding empty vectors to test for auto-activation and the levels were not any higher than the negative control (the 2^nd^ row). (E) Interaction between RctB and DnaK in vivo by the bacterial two hybrid (BATCH) assay. Here, as positive (+) control, a leucine zipper domain was fused to both the adenylate cyclase fragments T18 and T25 present in vectors pKT25 and pUT18C, respectively, and the clones were co-expressed. As negative control, the vectors were co-expressed (not shown). The cultures were spotted on MacConkey agar or LB+X-gal plates, and the intensities of colors (red or blue, respectively) were taken to indicate the strength of interactions. The negative control (pKT25+pUT18C) colors were similar to the spots shown for RctB+Vector (pUT18C) or DnaK+Vector (pKT25). Further details are in Materials and Methods.
