# Supplementary material for: The DnaK Chaperone Uses Different Mechanisms To Promote and Inhibit Replication of Vibrio cholerae Chromosome 2
Source: mBio. 2017 Apr 18;8(2):e00427-17. doi: 10.1128/mBio.00427-17 (PMC5395669; doi:10.1128/mBio.00427-17)
Supplement: FIG S2 [file mbo002173276sf2.docx]

**
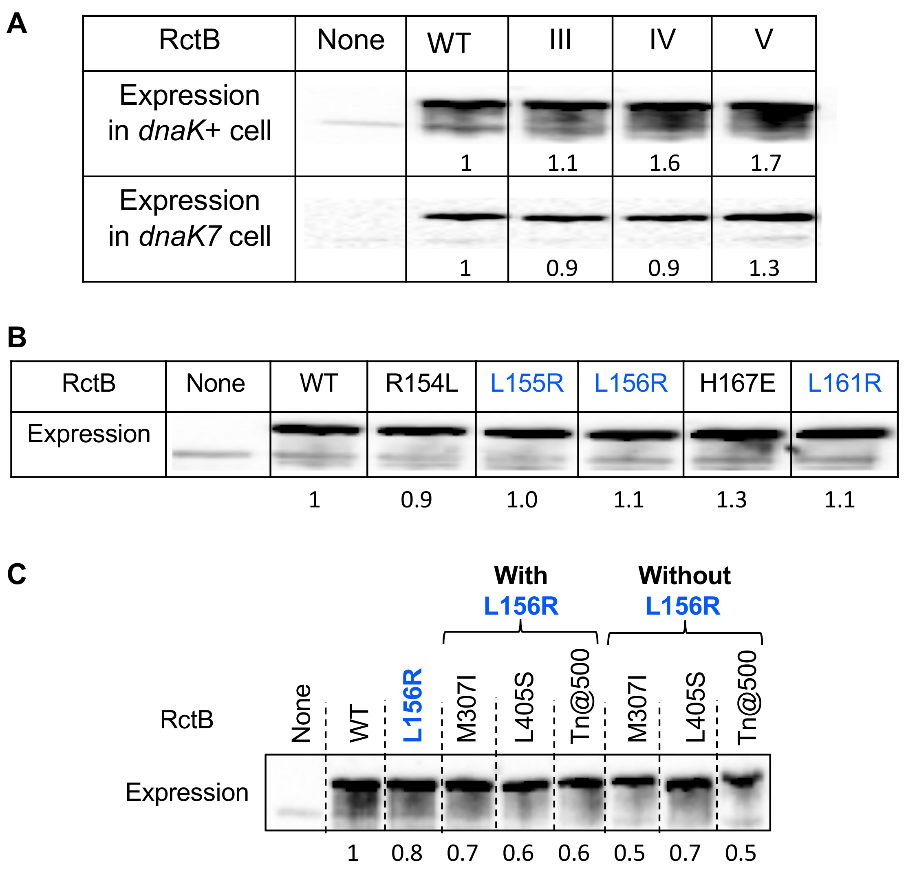
**

**Fig. S2. Expression levels of RctB mutants by Western blotting.** All Western blotting was performed following the protocol as described (1). The samples were from cultures used in Fig. 2B (**A**), Fig. 3B and C (**B**) and Fig. 4A to C (**C**). The relative protein expression levels are noted below the blots.

1. **Jha JK, Demarre G, Venkova-Canova T, Chattoraj DK.** 2012. Replication regulation of Vibrio cholerae chromosome II involves initiator binding to the origin both as monomer and as dimer. Nucleic Acids Res **40:**6026-6038.
