# Supplementary material for: The DnaK Chaperone Uses Different Mechanisms To Promote and Inhibit Replication of Vibrio cholerae Chromosome 2
Source: mBio. 2017 Apr 18;8(2):e00427-17. doi: 10.1128/mBio.00427-17 (PMC5395669; doi:10.1128/mBio.00427-17)
Supplement: FIG S3 [file mbo002173276sf3.docx]

**
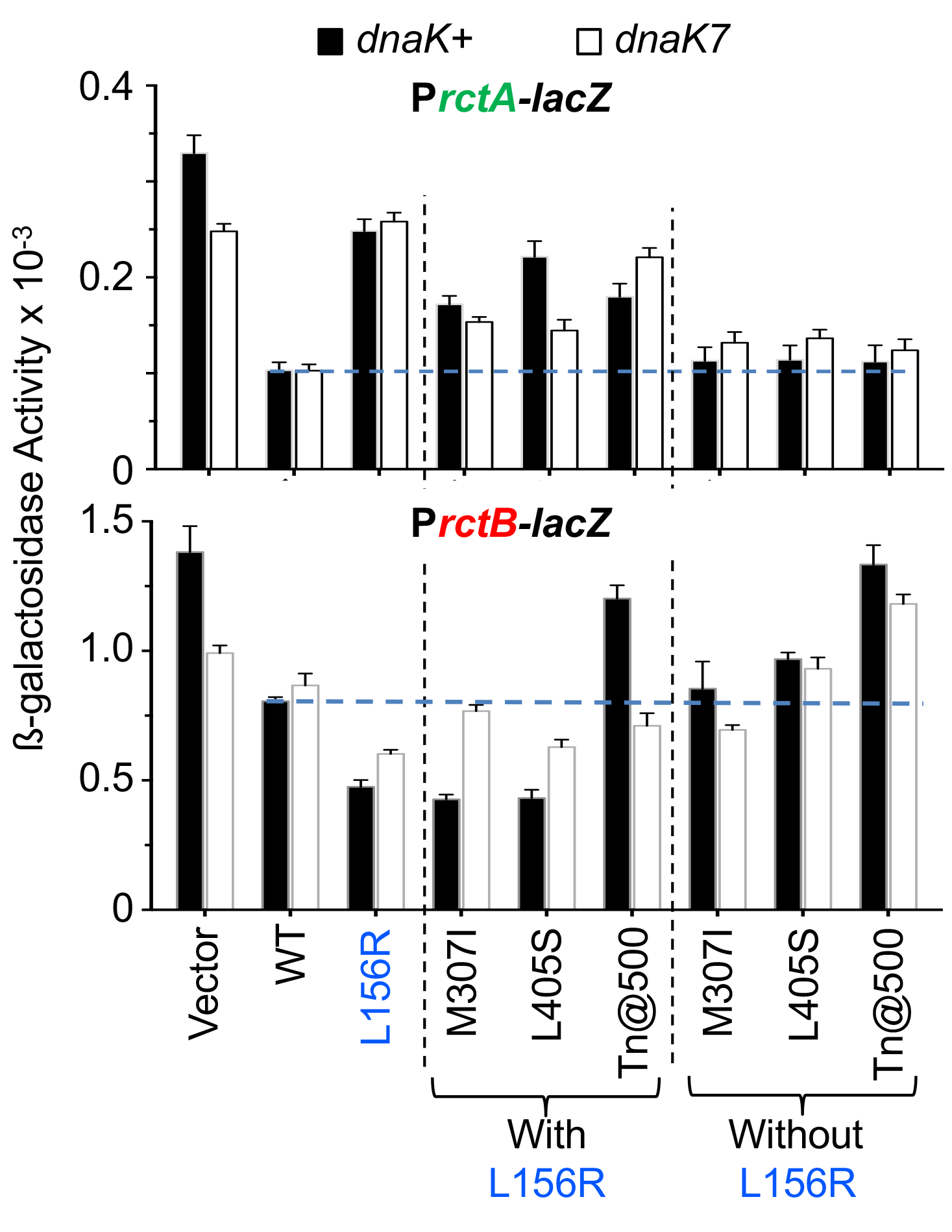
**

**Fig. S3. DNA binding of initiation‑proficient suppressors of initiation‑defective RctB mutant L156R in *dnaK*+ and *dnaK7* hosts.** The binding (promoter repression) data in the *dnaK*+ host (black bars) are from Fig. 4A and B. The promoter repression was measured similarly in the *dnaK7* host. The dashed line is provided as a visual aid for comparison of the activities with respect to the WT.
