# Supplementary material for: The DnaK Chaperone Uses Different Mechanisms To Promote and Inhibit Replication of Vibrio cholerae Chromosome 2
Source: mBio. 2017 Apr 18;8(2):e00427-17. doi: 10.1128/mBio.00427-17 (PMC5395669; doi:10.1128/mBio.00427-17)
Supplement: FIG S4 [file mbo002173276sf4.docx]

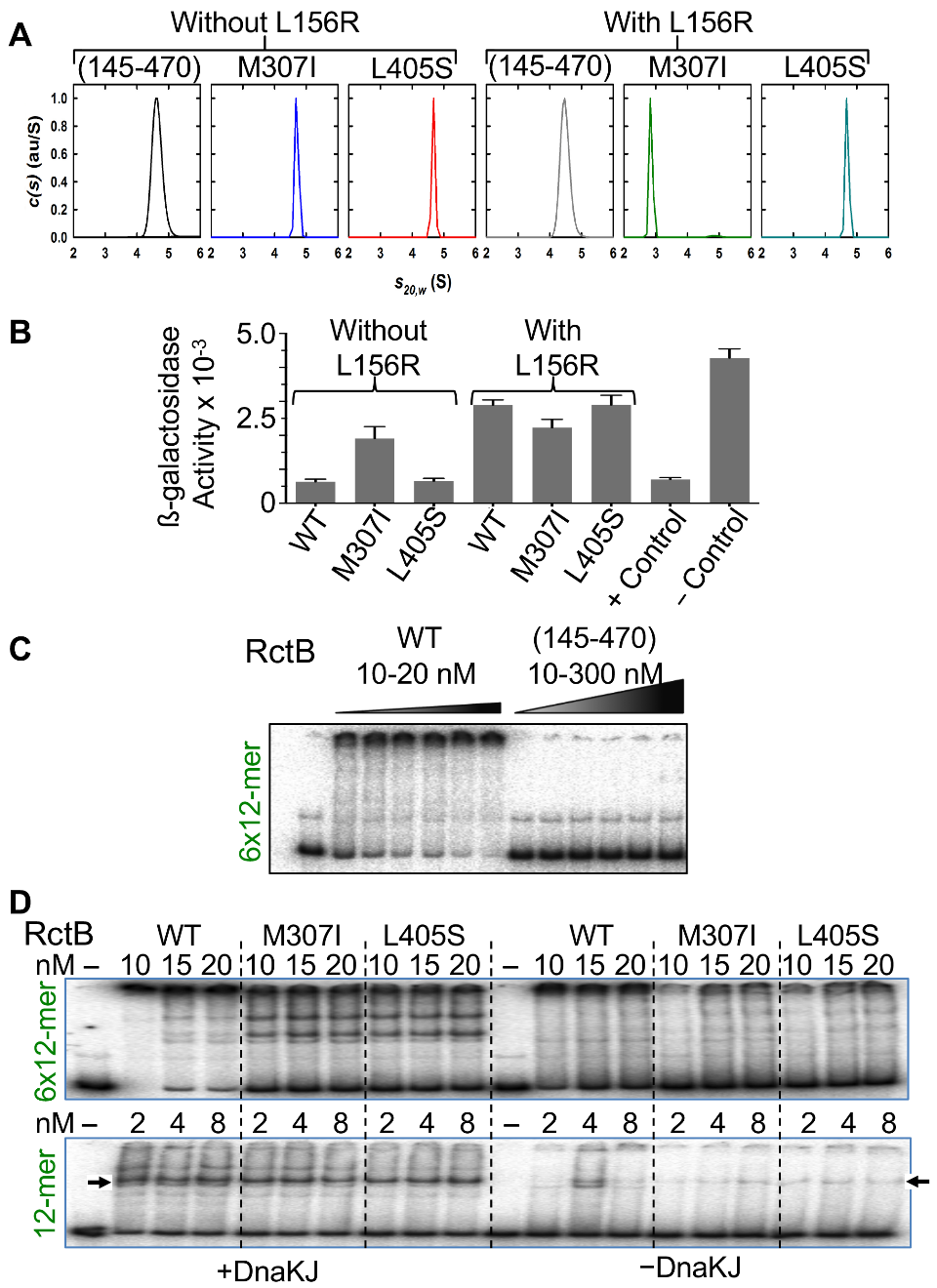
**Fig. S4. Dimerization and DNA binding activities of an RctB fragment with residues 145-470.** (**A**) Sedimentation velocity absorbance c(s) profiles for (from left to right) of MBP‑tagged RctB(145-470) fragment with substitutions and concentrations: none, 0.4 μM (black); M307I, 1.4 μM (blue); L405S, 1.2 μM (red); L156R, 0.7 μM (grey); L156R+M307I, 1.5 μM (green); and L156R+L405S, 1.6 μM (cyan). Sedimentation profile for RctB(145-470) (black) is consistent with the presence of a dimer at 4.64 S having a molar mass of 78 kDa. Similar observations were made for its blue and red derivatives. In the case of L156R (grey) and its cyan derivative, a single dimeric species is also observed at 4.49 S having a molar mass of 77 kDa; however, for the green derivative, a major species is now observed at 2.83 S. Based on the sedimentation coefficient and estimated mass of 37 kDa this represents a monomer. (**B**) Dimerization activity of RctB by the λcI repression assay *in vivo* with the substitutions tested in (A). The RctB used for fusion to λcIN was full-length. (**C**) EMSA of WT RctB and His‑RctB(145-470). RctB concentrations were 10, 12, 14, 16, 18 and 20 nM, and 10, 50, 100, 150, 200 and 300 nM in the two cases. (**D**) EMSA of full‑length MBP‑RctB carrying substitutions M307I and L405S (without the L156R change) in the presence and absence of DnaKJ. The arrow indicates monomer bound RctB. Note that the binding improves significantly in the presence of DnaKJ.
