# Supplementary material for: The DnaK Chaperone Uses Different Mechanisms To Promote and Inhibit Replication of Vibrio cholerae Chromosome 2
Source: mBio. 2017 Apr 18;8(2):e00427-17. doi: 10.1128/mBio.00427-17 (PMC5395669; doi:10.1128/mBio.00427-17)
Supplement: FIG S5 [file mbo002173276sf5.docx]

**
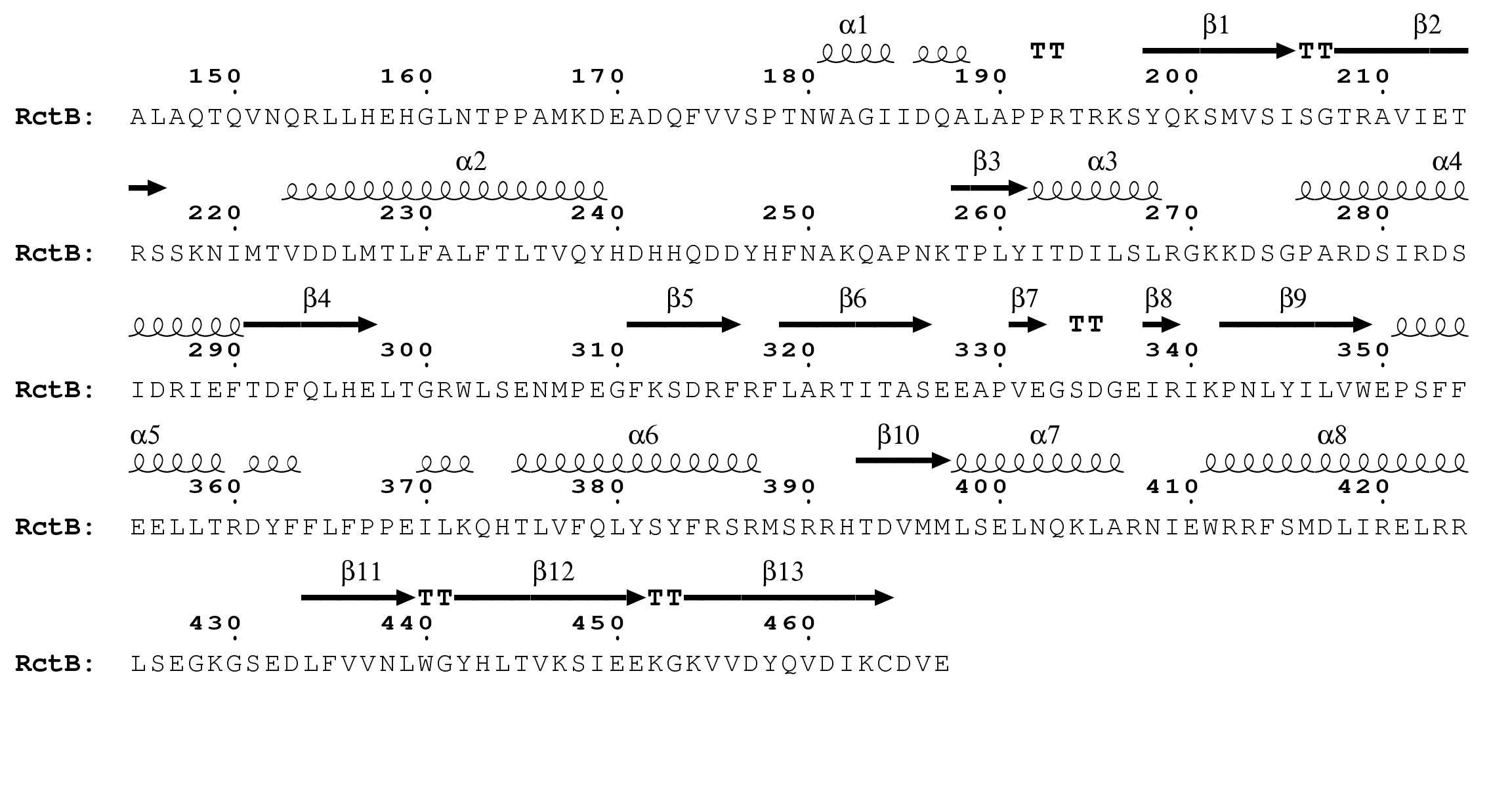
Fig. S5. Amino acid sequence and the secondary structure diagram of the tryptic fragment of RctB.** Helices and strands are marked and labeled. **TT** stands for turns.
