# Supplementary material for: The DnaK Chaperone Uses Different Mechanisms To Promote and Inhibit Replication of Vibrio cholerae Chromosome 2
Source: mBio. 2017 Apr 18;8(2):e00427-17. doi: 10.1128/mBio.00427-17 (PMC5395669; doi:10.1128/mBio.00427-17)
Supplement: FIG S6 [file mbo002173276sf6.docx]

**
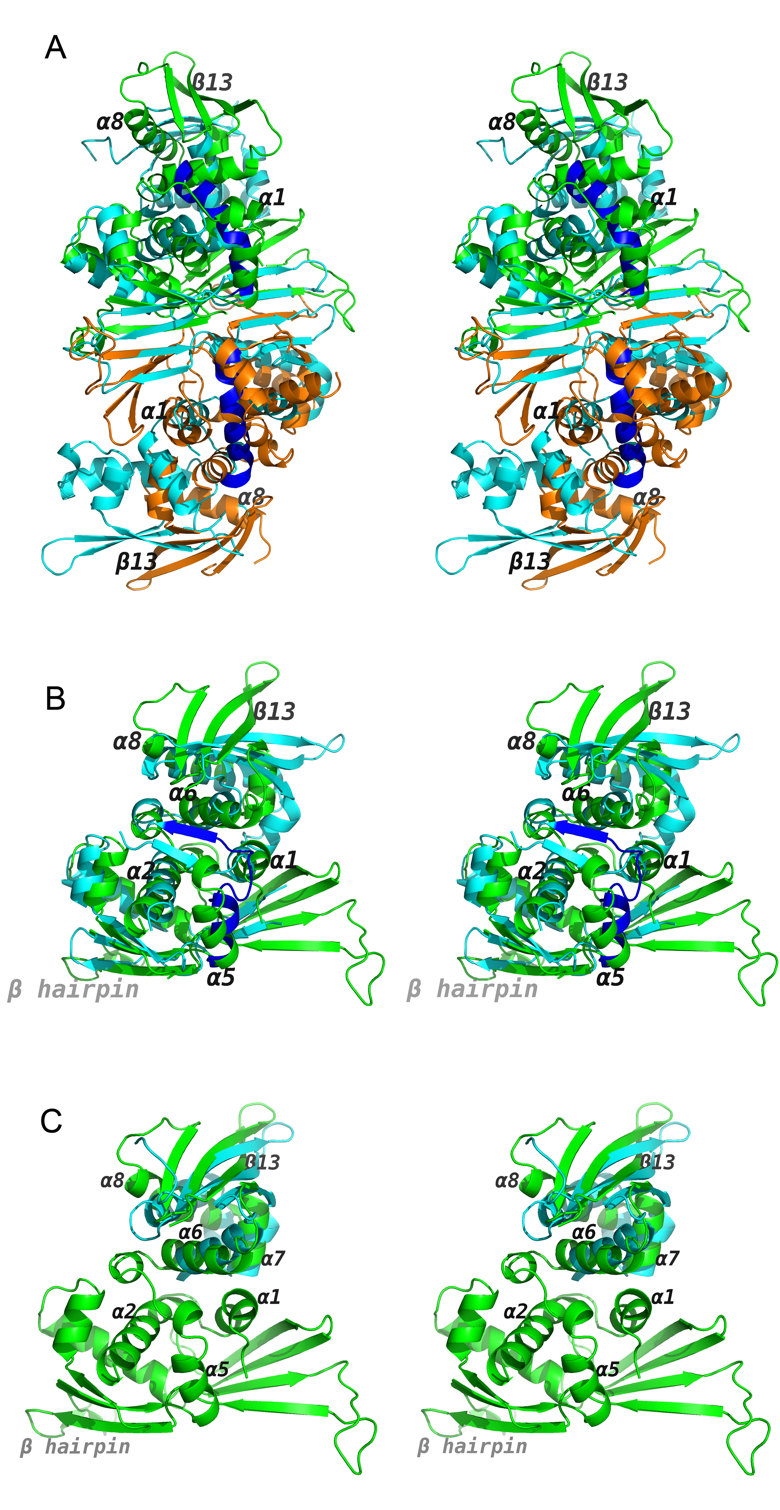
 Fig.** **S6.** **Superposition of the coordinates of RctB and RepE.** (**A**) Superposition of the RepE dimer on RctB. The two molecules in the RctB dimer are colored green and orange, whereas RepE dimer is colored cyan. Here and in panel B the equivalent of RctB α5 in RepE is dark blue. The C-domains are not superimposed (top of the figure). For clarity, only selected secondary structure elements are labeled. (**B**) Superposition of the C-terminal domain of RepE in its dimeric form (cyan) on the C-terminal domain of RctB (green), showing preservation of the fold. The N-terminal domain of RctB is green. In monomer to dimer conversion in RepE a long α5 helix of the dimer changes to a shorter α5 and a β strand (β1’ in Fig. 2 of (2)) (blue). The α5 in RctB is longer than in RepE monomer, but it is followed by a very short extended stretch followed by another helix that is very similar to the one in RepE monomer, but the dimer of RepE is very different. Thus RctB seems to have properties that are closer to the monomer, but the helix is almost as long as in the dimer. The significance of these changes remains to be understood. (**C**) Superposition of the C-terminal domain in a monomer of RepE (cyan) on the monomer of RctB (green), showing that both domains superpose well.

2. **Nakamura A, Wada C, Miki K.** 2007. Structural basis for regulation of bifunctional roles in replication initiator protein. Proc Natl Acad Sci U S A **104:**18484-18489.
