# Supplementary material for: The DnaK Chaperone Uses Different Mechanisms To Promote and Inhibit Replication of Vibrio cholerae Chromosome 2
Source: mBio. 2017 Apr 18;8(2):e00427-17. doi: 10.1128/mBio.00427-17 (PMC5395669; doi:10.1128/mBio.00427-17)
Supplement: FIG S7 [file mbo002173276sf7.docx]

**
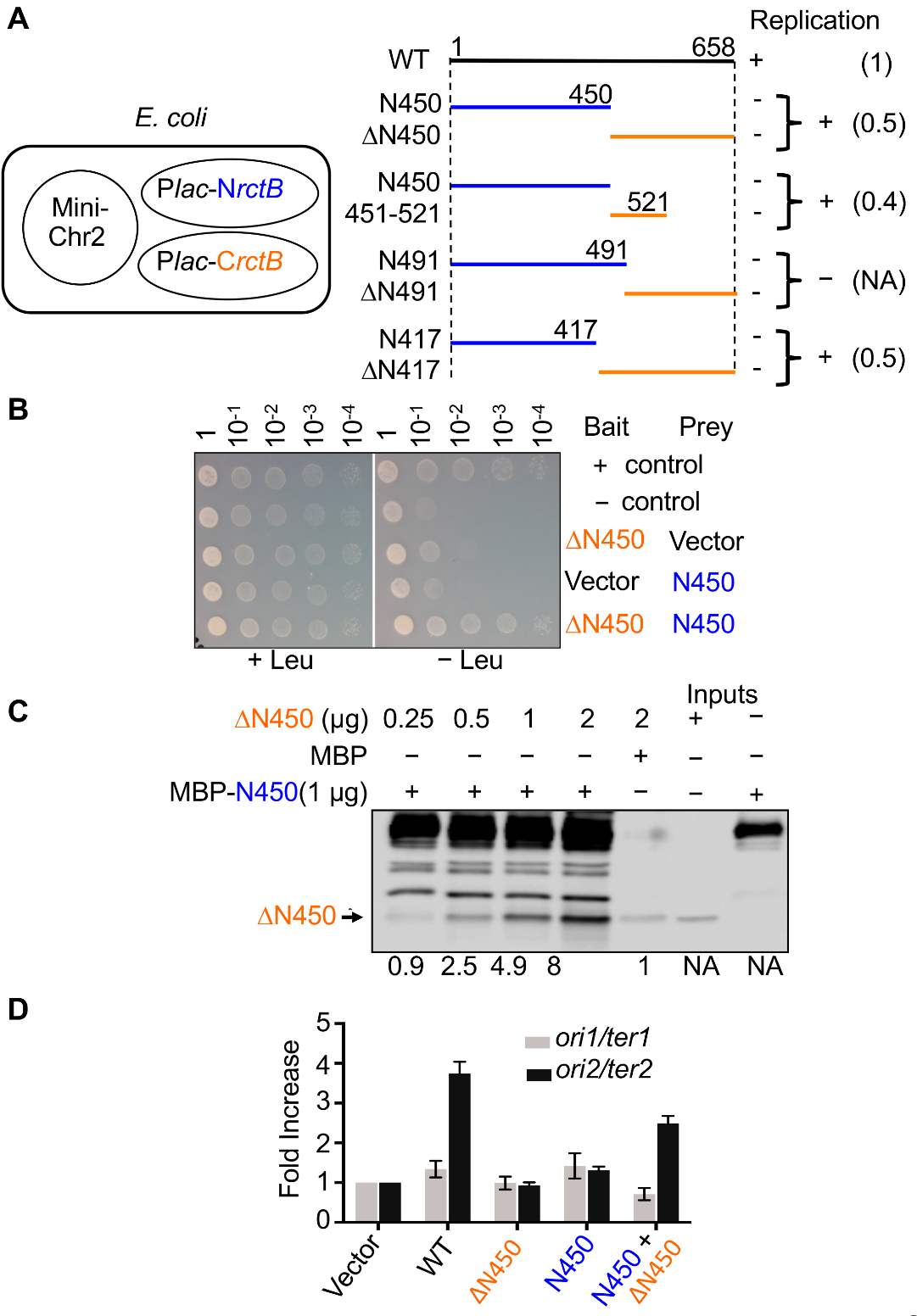
**

**Fig. S7. Interactions of the N- and C-terminal fragments of RctB.** (**A**) Complementation of the N‑ and C‑terminal fragments of RctB for initiator function in *E. coli*. The schematic on the left depicts the three‑plasmid system used for this assay. N-terminal fragments were tagged with the T18 fragment and the C-terminal fragments with the T25 fragment of the BATCH system. The fusions were used to stabilize the RctB fragments but were not essential (checked only for the N450 and ∆N450 pair). (**B**) Interaction by the Y2H assay. (**C**) Interaction by Co‑IP. (**D**) Stimulation of Chr2 replication in *V. cholerae* (N16961) by RctB fragments *in trans.* The *ori* and *ter* markers were measured by qPCR.
