# Supplementary material for: The DnaK Chaperone Uses Different Mechanisms To Promote and Inhibit Replication of Vibrio cholerae Chromosome 2
Source: mBio. 2017 Apr 18;8(2):e00427-17. doi: 10.1128/mBio.00427-17 (PMC5395669; doi:10.1128/mBio.00427-17)
Supplement: FIG S8 [file mbo002173276sf8.docx]

**
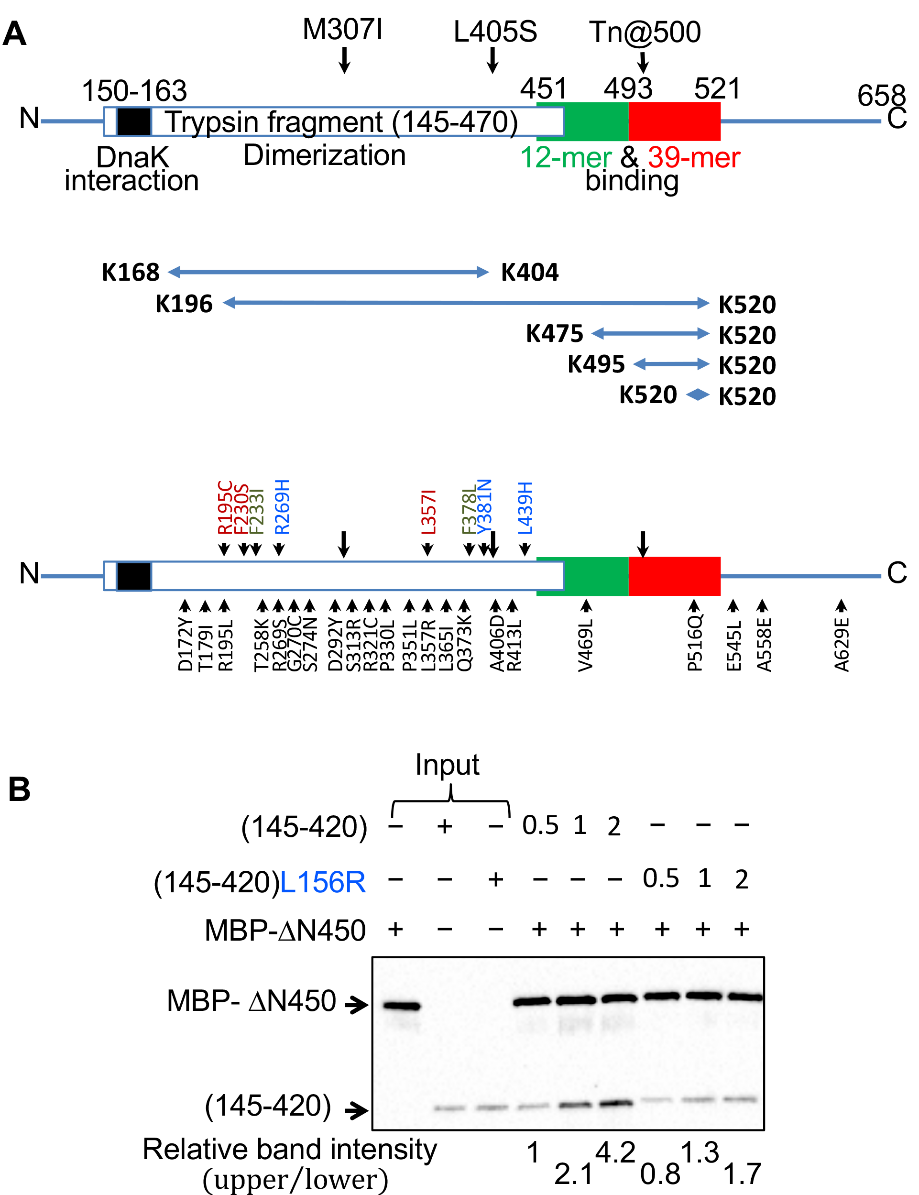
Fig. S8. Intra‑ and inter‑molecular interactions of RctB.** (**A**) Chemical cross-linking. The top diagram is a linear map of RctB marked with residue numbers of relevance to this study (not drawn to scale). The green and red boxes represent regions considered important for 12- and 39-mer binding, respectively (2) and shows the residue numbers of interest in the present study. The lysine residues identified to be cross-linked in the RctB dimer are shown by horizontal double arrows. When RctB(145-470) was used, only residues K168 and K404 were identified with reduced crosslinker adducts (the top double arrow). The remaining cross-linked residues were seen when analyzing full-length RctB. The bottom panel shows the positions (arrow heads) of previously characterized initiation‑proficient point mutants of RctB. Mutations in red are from (3), in green from (1), in blue from (4), and in black from (5). The long arrows are the suppressors isolated in the present study. (**B**) Interaction of RctB(145-420) and the fragment with the C-terminal 208 residues (∆N450) by Co‑IP. Note that RctB(145-420) is shorter than RctB(145-470) used earlier to avoid any overlap with ∆N450 fragment. The RctB(145-420) fragment was either WT or carried the L156R substitution. In the pulldown assay 1µg MBP-∆N450 protein was incubated with 0.5, 1 and 2 µg of RctB (145-420) or RctB (145-420) L156R in a reaction mixture (50 µl). Same pulldown and detection of the RctB procedure was followed as in Fig 1A. The $\mathrm{upper}/\mathrm{lower}$ values indicate the ratio of band intensities normalized to the value of MBP‑∆N450 (1 µg) and RctB(145-420) (0.5 µg) pair. Note that the normalized values decrease when the L156R substitution is present.

1. **Jha JK, Demarre G, Venkova-Canova T, Chattoraj DK.** 2012. Replication regulation of *Vibrio cholerae* chromosome II involves initiator binding to the origin both as monomer and as dimer. Nucleic Acids Res **40:**6026-6038.

2. **Jha JK, Ghirlando R, Chattoraj DK.** 2014. Initiator protein dimerization plays a key role in replication control of *Vibrio cholerae* chromosome 2. Nucleic Acids Res **42:**10538-10549.

3. **Val ME, Marbouty M, de Lemos Martins F, Kennedy SP, Kemble H, Bland MJ, Possoz C, Koszul R, Skovgaard O, Mazel D.** 2016. A checkpoint control orchestrates the replication of the two chromosomes of *Vibrio cholerae*. Sci Adv **2:**e1501914.

4. **Koch B, Ma X, Lφbner-Olesen A.** 2012. *rctB* mutations that increase copy number of *Vibrio cholerae oriCII* in *Escherichia coli*. Plasmid **68:**159-169.

5. **Yamaichi Y, Gerding MA, Davis BM, Waldor MK.** 2011. Regulatory cross-talk links *Vibrio cholerae* chromosome II replication and segregation. PLoS Genet **7:**e1002189.
