# Supplementary material for: The DnaK Chaperone Uses Different Mechanisms To Promote and Inhibit Replication of Vibrio cholerae Chromosome 2
Source: mBio. 2017 Apr 18;8(2):e00427-17. doi: 10.1128/mBio.00427-17 (PMC5395669; doi:10.1128/mBio.00427-17)
Supplement: TEXT S1 [file mbo002173276s1.docx]

**The DnaK Chaperone Uses Different Mechanisms to Promote and Inhibit Replication of *Vibrio cholerae* Chromosome 2**

**Jyoti K. Jha, Mi Li, Rodolfo Ghirlando, Lisa M. Miller Jenkins, Alexander Wlodawer, Dhruba Chattoraj**

**Supplemental materials**

**Text S1. Methods and Tables**

**Bacterial two-hybrid assay:** A bacterial adenylate cyclase two-hybrid (BACTH) system (EUK001; Euromedex, France) was used per the manufacturer’s protocol. The genes for the WT RctB and its N terminal deletion derivatives (∆N100 and ∆N200) were fused in frame to the C terminus of one of the adenylate cyclase fragments, T25, present in the vector pKT25 that resulted in plasmids, pBJH93, pJJ209 and pJJ210, respectively. The *E. coli* DnaK gene was fused in frame to the other adenylate cyclase fragment, T18, present in the vector pUT18C that created the plasmid, pJJ201 (Table S1). For cloning, the genes were amplified by PCR with Fusion polymerase (NEB) using primers listed in Table S2. Cloning in the pKT25 vector was done at PstI and BamHI sites, and in pUT18C at PstI and BamHi sites. Individual bait and prey plasmids were used together to transform E. coli BTH101 (CVC1837) cells, and the mixtures were grown overnight at 30°C in L broth with appropriate antibiotics in the presence of 0.5 mM Isopropyl-D thiogalactopyranoside (IPTG). The cultures were spotted on Luria agar containing 0.5 mM IPTG and 40 µg/ml of Xgal (5-bromo-4-chloro-3-indolyl-β-D-galactopyranoside) and on MacConkey agar containing same concentration of IPTG and appropriate antibiotics. Pictures were taken after 48 h of incubation at 30°C.

**Chemical crosslinking:** DTSSP is a water-soluble amine-reactive NHS-ester with a central disulfide bond that can be cleaved with DTT. Following crosslinking of RctB proteins with DTSSP, the protein was separated by SDS-PAGE to distinguish monomeric from dimeric forms. The monomeric and dimeric protein bands were separately cut and in-gel trypsin digested at 37°C for 16 h without reduction. The resultant peptides were then split, with one half being reduced with DTT and the other half left untreated. The peptides were then analyzed by liquid chromatography-mass spectrometry on an Orbitrap Fusion (Thermo) mass spectrometer. Both the MS1 and MS2 spectra were collected in the Orbitrap detector; the target resolution for each MS1 and MS2 spectrum was 60000 and 15000, respectively. Mass spectrometry data were analyzed using Proteome Discoverer 2.0 to check protein identification and convert raw data to MGF and StavroX (1) for analysis of sites of crosslinking.

**Pull down assay for N- and C-terminal fragments of RctB:** *In vitro* pull down assays were performed in a buffer A (20 mM Tris.HCl pH7.4, 100mM potassium glutamate, 0.1 mM EDTA, 1mM DTT, 0.1% Igepal CA-630, and 5% glycerol) taking 1µg of MBP-N450 and variable amount of ∆N450 RctB (ratio ranging 1:0.5 to 1:2) in a reaction mixture (50 µl). The mixtures were incubated for 30 min at 4°C with rocking and diluted 10-fold with the buffer B (50 mM Tris.HCl, pH 7.4, 100 mM NaCl, 0.1% Tween 20). Then, 20 µl slurry of amylose magnetic bead (NEB #E8035) in buffer B was added and incubated 2 h at 4°C with rocking. The magnetic beads were collected and washed 4 times with buffer C (50 mM Tris.HCl, pH 7.4, 300 mM NaCl, 0.2% Tween 20, and 0.1 mM EDTA). Bound proteins with the bead were eluted with 1X SDS‑PAGE loading buffer, ran on SDS-PAGE gel and transferred on nitrocellulose membrane. Following the transfer, RctB was detected with RctB polyclonal antibody as described before (2).

**Table S1. Bacterial strains and plasmids used in this study.**

| **Strains** | **Relevant characteristics** | **Source/Figure** |
| --- | --- | --- |
| CVC1797 | BR8706(λP*rctA-lacZ*); *dnaK+*; The fusion source was pTVC126 and transferred to λDKC331for chromosomal integration | Fig 2-4,6, Fig S3; (3) |
| CVC1798 | Same as CVC1797 except that the fusion is P*rctB-lacZ*, whose source was pTVC500 | „ |
|  |  |  |
| CVC2844 | Same fusion as CVC1797 except that the host was BR4390 (*dnaK7*) | Fig 2,3; Fig S3; This work & (4) |
| CVC2848 | Same fusion as CVC1798 except that the host was BR4390 (*dnaK7*) | „ |
| DH5α | *fhuA2* ∆(*lacZYA*-*argF*)U169 Φ80*dlacZ*∆M15 *gyrA96 recA1 relA1 endA1 thi-1 deoR hsdR17* | Invitrogen |
| BL21(λDE3) | Supplies T7 RNA polymerase from *lacUV5* promoter | Stratagene |
|  |  |  |
| **Plasmids** |  |  |
| pTVC16 | P_T7_-*rctB* WT in pTXB1; pBR*ori*; Ap^R^ | Fig S1A; (2) |
| pMAL-c2X | Expression of MBP and MBP-tagged proteins; pBR*ori*; Ap^R^ | Fig 1A,C; Fig S1 A-C; NEB |
| pJJ56 | P*tac*-*rctB*WT (aa 1-658) in pMAL-c2X; pBR*ori*; Ap^R^ | Fig 1A,C,2A,3A;  Fig S1 A‑C |
| pJJ181 | Same as pJJ56 except for *rctB*∆N10 (aa 11-658) | Fig S1C |
| pJJ182 | „ *rctB*∆N20 (aa 21-658) | „ |
| pJJ183 | „ *rctB*∆N50 (aa 51-658) | „ |
| pJJ184 | „  *rctB*∆N100 (aa 101-658) | Fig S1C; Fig 1A |
| pJJ185 | „  *rctB*∆N200 (aa 201-658) | „ ; „ |
| pJJ194 | „  *rctB*∆N400 (aa 401-658) | Fig S1C |
| pJJ187 | „ *rctB*∆N120 (aa 121-658) | Fig 1A |
| pJJ188 | „ *rctB*∆N140 (aa 141-658) | „ |
| pJJ189 | „ *rctB*∆N160 (aa 161-658) | „ |
| pJJ190 | „  *rctB*∆N180 (aa 181-658) | „ |
| pJJ191 | „  *rctB*∆N450 (aa 451-658) | Fig S1B, Fig S7B |
|  |  |  |
| pJJ428 | „ *rctB*I (LVTESVK → 7xA) | Fig 1C |
| pJJ429 | „ *rctB*II (QNEKQKR → 7xA) | „ |
| pJJ208 | „ *rctB*III (SKALAQT → 7xA) | Fig 1C, 2A |
| pJJ231 | „ *rctB*IV (QVNQRLL → 7xA) | „ , „ |
| pJJ229 | „ *rctB*V (HEHGLNT → 7xA) | „ , „ |
| pJJ430 | „ *rctB*VI (PPAMKDE → 7xA) | Fig 1C |
|  |  |  |
| pSH18-34 | LRI∆1 derivative with eight *lexA*op controlling *lacZ* reporter*;* pBR*ori*, 2μ*ori*; Ap^R^, *URA3* | Fig 1D, Fig S1D; Clontech |
| pSH17-34 | LexA-GAL4 activation domain fusion under *ADH* promoter; positive control; 2μ*ori*; Ap^R^, *HIS3* | „ |
| pRFHM1 | LexA-bicoid homeodomain fusion under *ADH* promoter; negative control; 2μ*ori*; Ap^R^, *HIS3* | „ |
| pEG202 | Bait vector in yeast two-hybrid system; pBR*ori*, 2μ*ori*; Ap^R^, *HIS3* | „ |
| pJG4-5 | Prey vector in yeast two-hybrid system; pUC*ori*, 2μ*ori*; Ap^R^, *TRP1* | „ |
| pJJ218 | *rctB* WT in pEG202 | Fig 1D, Fig S1D |
| pJJ223 | *dnaK_Vc_* in pJG4-5 | „ |
| pJJ253 | *rctB*III (SKALAQT → 7xA) in pEG202 | Fig 1D |
| pJJ254 | Same as pJJ253 except for *rctB*IV (QVNQRLL → 7xA) | „ |
| pJJ255 | „ *rctB*V (HEHGLNT → 7xA) | „ |
| pJJ224 | „ *rctB*∆N100 (aa 101-658) | Fig S1D |
| pJJ225 | „ *rctB*∆N200 (aa 201-658) | „ |
| pJJ221 | *rctB*∆N450 in pEG202 | Fig S7B |
| pJJ222 | *rctB*N450 in pJG4-5 | „ |
|  |  |  |
| pJJ114 | Mini-Chr2 (same as pTVC31 except for the drug marker), contains *ori2* (nt 775–1133); R6K*ori***γ**; Cm^R^ | Fig 1E, 3C, 4C, 6C; (5) |
|  |  |  |
| pET22b | Expression of 6xH-tagged proteins; pBR*ori*; Ap^R^ | Fig 2B,3B,3C,4,6;  Novagen |
| pTVC14 | P_T7_-*rctB* WT-6xH in pET22b; pBR*ori*; Ap^R^ | Fig 1E,2B,3B, 3C,4,6, Fig S3; (6) |
| pJJ240 | Same as pTVC14 except for *rctB*I (HEHGLNT → 7xA) | Fig 1E |
| pJJ241 | „ *rctB*II (QNEKQKR → 7xA) | „ |
| pJJ242 | „ *rctB*III (SKALAQT → 7xA) | Fig 1E, 2B |
| pJJ243 | „ *rctB*IV (QVNQRLL → 7xA) | „ |
| pJJ244 | „ *rctB*V (HEHGLNT → 7xA) | „ |
| pJJ343 | „ *rctB*VI (PPAMKDE → 7xA) | Fig 1E |
|  |  |  |
| pJJ56 | P*tac*-*rctB*WT (aa 1-658) in pMAL-c2X; pBR*ori*; Ap^R^ | Fig 3A |
| pJJ257 | Same as pJJ56 except for *rctB* (R154L) | „ |
| pJJ259 | „ *rctB* (L155R) | „ |
| pJJ258 | „ *rctB* (L156R) | „ |
| pJJ265 | „ *rctB* (H157E) | „ |
| pJJ266 | „ *rctB* (L161R) | „ |
|  |  |  |
| pTVC14 | P_T7_-*rctB* WT-6XH in pET22b; pBR*ori*; Ap^R^ | Fig 3B, 3C |
| pJJ262 | Same as pTVC14 except for *rctB* (R154E) | „ |
| pJJ260 | „ *rctB* (L155R) | „ |
| pJJ263 | „ *rctB* (L156R) | „ |
| pJJ261 | „ *rctB* (H157E) | „ |
| pJJ264 | „ *rctB* (L161R) | „ |
|  |  |  |
| pJJ394 | „ *rctB* (M307I) | Fig. 4, S3 |
| pJJ395 | „ *rctB* (L405S) | „ |
| pJJ409 | „ *rctB* (Tn@500) | „ |
|  |  |  |
| pBJH93 | T25-*rctB*WT (aa 1-658) in pKT25; p15A*ori*; Kn^R^ | Fig S7A ; (7) |
| pJJ79 | P*lac*-T18-*rctB*N450; pUC*ori*; Ap^R^ | Fig S7A |
| pJJ81 | P*lac*-T25-*rctB* ∆N450 (aa 451- 658); p15A*ori*; Kn^R^ | „ |
| pJJ216 | P*lac*-T25-*rctB* (aa 451-521); p15A*ori*; Kn^R^ | „ |
| pJJ172 | P*lac*-T18-*rctB*N491; pUC*ori*; Ap^R^ | „ |
| pJJ174 | P*lac*-T25-*rctB*∆N491; p15A*ori*; Kn^R^ | „ |
| pJJ177 | P*lac*-T18-*rctB*N417; pUC*ori*; Ap^R^ | „ |
| pJJ195 | P*lac*-T25-*rctB*∆N417; p15A*ori*; Kn^R^ | „ |
|  |  |  |
|  |  |  |
| pJJ112 | P*lac*-λcIN-*rctB*WT; pBR*ori*; Ap^R^ | Fig 6; Fig S4 |
| pJJ414 | Same as pJJ112 except for *rctB*M307I | Fig S4B |
| pJJ392 | „  *rctB*L405S | „ |
| pJJ417 | „ *rctB*L156R | „ |
| pJJ432 | Same as pJJ417 except for *rctB*L156R+M307I | „ |
| pJJ433 | „ *rctB*L156R+L405S | „ |
|  |  |  |
| pJJ462 | Same as pJJ112 except for *rctB* (G310P) | Fig 6B |
| pJJ463 | „ *rctB* (F311P) | „ |
| pJJ464 | „ *rctB* (K312P) | „ |
| pJJ465 | „ *rctB* (S313P) | „ |
| pJJ466 | „ *rctB* (D314P) | „ |
| pJJ467 | „ *rctB* (R315P) | „ |
| pJJ468 | „ *rctB* (F316P) | „ |
| pJJ469 | „ *rctB* (R317P) | „ |
|  |  |  |
| pET28a | Expression of 6xH-tagged proteins; pBR*ori*; Kn^R^ | Novagen |
| **pJJ316** | P_T7_-*rctB* (145-470)-6xH in pET28a | Fig S4A |
| pJJ458 | Same as pJJ316 except for *rctB*(145-470)M307I | „ |
| pJJ459 | „ *rctB* (145-470)L405S | „ |
| **pJJ399** | P_T7_-*rctB* (145-470)L156R-6xH in pET28a | „ |
| pJJ460 | Same as pJJ399 except for *rctB*(145-470)L156R+M307I | „ |
| pJJ461 | „ *rctB* (145-470)L156R+L405S | „ |
|  |  |  |
| pJJ56 | P*tac*-*rctB*WT in pMAL-c2X; pBR*ori*; Ap^R^ | Fig S4D |
| pJJ397 | „ *rctB* (M307I) | „ |
| pJJ398 | „  *rctB* (L405S) | „ |
|  |  |  |
| pUT18C‑zip | Bacterial two‑hybrid positive control | Fig S1E; Euromedex |
| pKT25‑zip | Bacterial two‑hybrid positive control | „ ; „ |
| pKT25 | Bacterial two‑hybrid bait vector; p15A*ori*; Kn^R^ | „ ; „ |
| pUT18C | Bacterial two‑hybrid pray vector; pUC*ori*; Ap^R^ | „ ; „ |
| pBJH93 | T25-*rctB*WT (aa 1-658) in pKT25; p15A*ori*; Kn^R^ | „ ; (7) |
| pBJH95 | T18-*rctB*WT (aa 1-658) in pUT18C | „ ; „ |
| pJJ200 | T25-*dnaK_Ec_* in pKT25 | „ |
| pJJ201 | T18-*dnaK_Ec_* in pUT18C | „ |
| pJJ209 | T25-*rctB*∆N100 (aa 101-658) in pKT25 | „ |
| pJJ210 | T25-*rctB*∆N200 (aa 201-658) in pKT25 | „ |
|  |  |  |
|  |  |  |
| pTVC14 | P_T7_-*rctB* WT-6XH in pET22b; pBR*ori*; Ap^R^ | Fig 6C |
| pJJ443 | Same as pTVC14 except for *rctB* (G310P) | „ |
| pJJ444 | „ *rctB* (F311P) | „ |
| pJJ445 | „ *rctB* (K312P) | „ |
| pJJ446 | „ *rctB* (S313P) | „ |
| pJJ437 | „ *rctB* (D314P) | „ |
| pJJ447 | „ *rctB* (R315P) | „ |
| pJJ448 | „ *rctB* (F316P) | „ |
| pJJ449 | „ *rctB* (R317P) | „ |
|  |  |  |
| pJJ56 | P*tac*-*rctB*WT in pMAL-c2X; pBR*ori*; Ap^R^ | Fig 7A |
| pJJ451 | Same as pJJ56 except for *rctB* (F311P) | „ |
| pJJ452 | „ *rctB* (K312P) | „ |
| pJJ453 | „ *rctB* (F313P) | „ |
| pJJ456 | „ *rctB* (F316P) | „ |
|  |  |  |
| pTVC174 | 39-mer (nt 449–487) in pBEND2; Ap^R^ | Fig 2,3,7; Fig S4 |
| pTVC195 | 1x12-mer (nt 565–602) in pBEND2; Ap^R^ | Fig 7 ; „ |
| pTVC228 | 6x12-mer (nt 788–934) in pTVC243; Cm^R^ | Fig 2,3,7; „ |
|  |  |  |

**Table S2. Primers used in this study**

| Name | Sequence (5′-3′) | Purpose  (Plasmid Construction) |
| --- | --- | --- |
| jj23 | ATCACTCGAGTTAGGCTCCAGCGGCCATCTC | pJJ181-185,187-190, 208,218,221,224,225,229,231,253-255, 430,428,429 |
| jj51 | AGTAGGATCCTTACTGCTTGGAAACCAGCTC | pJJ216 |
| jj60 | CGTCCATATGAGCTCAGAAGAAAAACGA | pJJ240-244,343 |
| jj70 | AGTAGGATCCTTAGGCTCCAGCGGCCATCTC | pJJ |
| jj80 | ATCACTCGAGGGCTCCAGCGGCCATCTCAT | pJJ240-244,343 |
| jj113 | ATCACTCGAGTTCAATGCTTTTCACAGTC | pJJ191,222 |
| jj115 | ATGCGAATTCATGAGCTCAGAAGAAAAACG | pJJ191,208, 218, 222, 229,231,253-255,430,428,429 |
| jj131 | ATGCCTGCAGGATGAGCTCAGAAGAAAAACGA | pJJ79,177,178,179, 214, 215, 216 |
| jj132 | ATCAGGATCCTTCAATGCTTTTCACAGTC | pJJ79,80 |
| jj133 | ATGCCTGCAGGGGAGAAAGGCAAAGTGGTGGA | pJJ81,216,211 |
| jj134 | AGTAGGATCCGGCTCCAGCGGCCATCTCAT | pJJ81,195, 209,210,212,213 |
| jj135 | ATGCCTGCAGGGAGAAAGGCAAAGTGGTGGA | pJJ78 |
| jj136 | ATGCCTGCAGGGATGAGCTCAGAAGAAAAACGA | pJJ80 |
| jj128 | TTCTTCTGAGCTCATATGTAGATCCGTATCAGCTAGCC | pJJ(69-75), 87 |
| jj129 | ATGTTCTAGAAAGCTTGGCTGCAGGTCGATG | pJJ(69-75), 87 |
| jj197 | AGTCCATATGGCCGCAGCAGCAGCACGATTGATCAAATTGCC | pJJ131 |
| jj23 | ATCACTCGAGTTAGGCTCCAGCGGCCATCTC | pJJ131,132,193,194, 218,221, 224,225 |
| jj198 | AGTCCATATGAGCTCAGAAGAAAAAGCAGCGGCCGCAGCGCCA AGAACTCACAAAGATG | pJJ132 |
| jj224 | TGCCATATGAGCTCAGAAGAAAAACGATTGATCAAATTG GCAGCAGCTGCCGCAGATGGTCATCTTTTTGA | pJJ143 |
| jj080 | ATCACTCGAGGGCTCCAGCGGCCATCTCAT | pJJ38,39,44,67,86,134,135,143 |
| jj256 | ATCCATAGAAAACCGTCGCCATTCG | pJJ177 |
| jj257 | AAATAGGAGTAGAGCTGAAATACCAAG | pJJ178 |
| jj259 | GCGAAAACGATCGCTTTTAAAGCCTTC | pJJ179 |
| jj276 | ATGCCTGCAGGGCTGATCCGCGAACTTCGTC | pJJ195 |
| jj282 | CGCTGCTGCTGCTGCGCGCTTTTGCTTTTCATTTTGC | pJJ208 |
| jj161 | CGTCCATATGCATCTTTTTGAAGTCTCTGAAGC | pJJ125 |
| jj162 | CGTCCATATGGGATTACGCAGTAGAGATGGC | pJJ126 |
| jj78 | GATCCATATGCGTTTTGAAGAGGGGCTG | pJJ127 |
| jj66 | AGTCCATATGGAGAAAGGCAAAGTGGTGGA | pJJ 153 |
| jj250 | GCCAGGATCC G ATGAGCTCAGAAGAAAAACG | pJJ 154,155 |
| jj251 | ATCAGCGGCCGCTTCAATGCTTTTCACAGTC | pJJ 154,155 |
| jj256 | ATCCATAGAAAACCGTCGCCATTCG | pJJ177 |
| jj261 | CGTCGAATTCATGTTGCCAAGAACTCACAAAGA | pJJ181 |
| jj262 | CGTCGAATTCATGCATCTTTTTGAAGTCTCTGAAG | pJJ182 |
| jj263 | CGTCGAATTCATGCTGATCTCACTGCGTGGATTAC | pJJ183 |
| jj264 | CGTCGAATTCATGTTTGAAGAGGGGCTGGCTGGC | pJJ184,224 |
| jj265 | CGTCGAATTCATGTCTATGGTTTCGATATCGGGT | pJJ185,225 |
| jj266 | CGTCGAATTCATGATCTCGGTACTCGGCTCAAC | pJJ187 |
| jj267 | CGTCGAATTCATGAAGCGCTCAAAAGCATTAGC | pJJ188 |
| jj268 | CGTCGAATTCATGTTAAATACACCGCCAGCCAT | pJJ189 |
| jj269 | CGTCGAATTCATGTGGGCAGGGATCATTGATC | pJJ190 |
| jj271 | AATTCAAGCGCTCAAAAGCATTAGCGCAGACGCAAGTCAATCAACGTTTACTGCATGAGCATGGTTG | pJJ196 |
| jj272 | TCGACAACCATGCTCATGCAGTAAACGTTGATTGACTTGCGTCTGCGCTAATGCTTTTGAGCGCTTG | pJJ196 |
| jj273 | AATTCAAGCGC GCA GCA GCA GCA GCG GCG GCGCAAGTCAATCAACGTTTACTGCATGAGCATGGTTG | pJJ197 |
| jj274 | TCGACAACCATGCTCATGCAGTAAACGTTGATTGACTTGCGCCGCCGCTGCTGCTGCTGCGCGCTTG | pJJ197 |
| jj275 | GCAAAAGCGCGCAGCAGCAGCAGCGGCGGCGCAAGTCAATCAACGTTTAC | pJJ208 |
| jj276 | ATGCCTGCAGGGCTGATCCGCGAACTTCGTC | pJJ195 |
| jj277 | CGTCGAATTCATGCGTTGGCTCAGTGAGAATA | pJJ193 |
| jj278 | CGTCGAATTCATGCTCAACCAAAAATTGGCC | pJJ194 |
| jj279 | ATGCCTGCAGGGATGGGTAAAATAATTGGTATC | pJJ200 |
| jj280 | CGCAGGATCCTTTTTTGTCTTTGACTTCTTC | pJJ200, 201 |
| jj281 | ATGCCTGCAGGATGGGTAAAATAATTGGTATC | pJJ201 |
| jj282 | CGCTGCTGCTGCTGCGCGCTTTTGCTTTTCATTTTGC | pJJ208 |
| jj290 | ATGCCTGCAG GG ATGTTTGAAGAGGGGCTGGCTGG | pJJ209,224 |
| jj291 | ATGCCTGCAG GG ATGTCTATGGTTTCGATATCGG | pJJ210,225 |
| jj292 | ATGCCTGCAGGGTTTTTAGCGCGCACCATCACCG | pJJ211 |
| jj293 | ATGCCTGCAGGGTTACGTGGCAAAAAAGACA | pJJ212 |
| jj294 | ATGCCTGCAGGGAAAAACATCATGACGGTCG | pJJ213 |
| jj295 | ATGCGGATCCAGAGAGAATGTCGGTGATATAC | pJJ214 |
| jj296 | ATGCGGATCCAGACGATCGTGTTTCAATC | pJJ215 |
| jj301 | ACGCGAATTCGAGAAAGGCAAAGTGGTGG | pJJ221 |
| jj302 | ACGCGTCGACATGGGTAAAATCATTGGTATTG | pJJ223 |
| jj303 | ATGTGTCGACTTACTTCTTATCGTCGTTAAC | pJJ223 |
| jj308 | GCAGCAGCAGCAGCGGCGGCG CAAAATGAAAAGCAAAAGC | pJJ428 |
| jj309 | CGCCGCCGCTGCTGCTGCTGC GCTGGTTGAGCCGAGTAC | pJJ428 |
| jj310 | GCA GCA GCA GCA GCG GCG GCG CATGAGCATGGTTTAAATAC | pJJ231 |
| jj311 | CGC CGC CGC TGC TGC TGC TGC CGTCTGCGCTAATGCTTTTG | pJJ231 |
| jj312 | GCAGCAGCAGCAGCGGCGGCGACACCGCCAGCCATGAAAG | pJJ229 |
| jj313 | CGCCGCCGCTGCTGCTGCTGCTAAACGTTGATTGACTTGC | pJJ229 |
| jj332 | CAA GTC AAT CAA GAA TTA CTG CAT GAG CAT GG | pJJ257,262 |
| jj333 | CTCATGCAGTAATTCTTGATTGACTTGCGTCTG | pJJ257,262 |
| jj334 | GTC AAT CAA CGT CGA CTG CAT GAG CAT GGT T | pJJ259,260 |
| jj335 | TGCTCATGCAGTCGACGTTGATTGACTTGCG | pJJ259,260 |
| jj336 | C AAT CAA CGT TTA CGG CAT GAG CATGGT TTA AAT AC | pJJ258,263,399,417 |
| jj337 | CCATGCTCATGCCGTAAACGTTGATTGACTTG | pJJ258,363,399,417 |
| jj338 | CAA CGT TTA CTG GAG GAG CAT GGT TTA AAT AC | pJJ261,265 |
| jj339 | TAAACCATGCTCCTCCAGTAAACGTTGATTG | pJJ261,265 |
| jj340 | CAT GAG CATGGT CGA AAT ACA CCG CCA GCC ATG | pJJ264,266 |
| jj341 | GGCGGTGTATTTCGACCATGCTCATGCAGTAAAC | pJJ264,266 |
| jj405 | CGTCCATATGGCATTAGCGCAGACGCAAGTC | pJJ316 |
| jj407 | ATCACTCGAG*TTA*GATCAGATCCATAGAAAACC | pJJ316 |
| jj514 | CAGTGAGAATATACCAGAAGGCTTTAAAAGCG | pJJ394,414,432,397 |
| jj515 | AGCCTTCTGGTATATTCTCACTGAGCCAACG | pJJ394,414,432,397 |
| jj516 | CTCAACCAAAAATCGGCCAGAAACATCGAATGG | pJJ392,395,433,398 |
| jj517 | GATGTTTCTGGCCGATTTTTGGTTGAGTTCACTC | pJJ392,395,433,398 |
| jj557 | GGCTTTAAAAGCCCTCGTTTTCGCTTTTTAGCG |  |
| jj575 | GGCTTTAAAAGCCCTCGTTTTCGCTTTTTAGCG | pJJ437,466 |
| jj576 | GCGAAAACGAGGGCTTTTAAAGCCTTCTGGC | pJJ437,466 |
| jj577 | GAATATGCCAGAACCCTTTAAAAGCGATCGTTTTC | pJJ443,451,462 |
| jj578 | GCTTTTAAAGGGTTCTGGCATATTCTCACTG | pJJ443,451,462 |
| jj579 | GCCAGAAGGCCCTAAAAGCGATCGTTTTCG | pJJ444,452,463 |
| jj580 | GATCGCTTTTAGGGCCTTCTGGCATATTCTC | pJJ444,452,463 |
| jj581 | GCCAGAAGGCTTTCCAAGCGATCGTTTTCGCT | pJJ445,453,464 |
| jj582 | CGATCGCTTGGAAAGCCTTCTGGCATATTC | pJJ445,453,464 |
| jj583 | AGGCTTTAAACCCGATCGTTTTCGCTTTTTAG | pJJ446,465 |
| jj584 | CGAAAACGATCGGGTTTAAAGCCTTCTGGCATAT | pJJ446,465 |
| jj585 | TAAAAGCGATCCTTTTCGCTTTTTAGCGCG | pJJ447,467 |
| jj586 | AAAGCGAAAAGGATCGCTTTTAAAGCCTTC | pJJ447,456,467 |
| jj587 | AAGCGATCGTCCTCGCTTTTTAGCGCGCACC | pJJ448,456,468 |
| jj588 | CTAAAAAGCGAGGACGATCGCTTTTAAAGCC | pJJ448,468 |
| jj589 | CGATCGTTTTCCCTTTTTAGCGCGCACCATC | pJJ449,469 |
| jj590 | CGCTAAAAAGGGAAAACGATCGCTTTTAAAG | pJJ449,469 |

**Table S3.** **Data collection and structure refinement**

| **Data collection** |  |
| --- | --- |
|  | SeMet RctB |
| Space group | *P*3_2_21 |
| Molecules/a.u. | 2 |
| Unit cell *a*, *b*, *c* (Å);  β (°) | 105.53, 105.53, 137.76,  90, 90, 120 |
| Resolution (Å) | 100.0-3.00 (3.11-3.00) |
| *R*_merge_^†^ (%) | 10.5 (66.6) |
| No. of reflections  (measured/unique) | 121359/18054 |
| <*I* / σ*I*> | 19.68 (1.64) |
| Completeness (%) | 99.1 (99.2) |
| Redundancy | 6.7 (6.2) |
|  |  |
| **Refinement** |  |
| Resolution (Å) | 43.37-3.00 |
| No. of reflections  (refinement/*R*_free_) | 17127/772 |
| *R* / *R*_free_^‡^ | 0.219/0.278 |
| No. atoms |  |
| Protein | 4558 |
| Ligands | 0 |
| Water | 0 |
|  |  |
| R.m.s. deviations from ideal values |  |
| Bond lengths (Å) | 0.018 |
| Bond angles (°) | 1.45 |
| PDB accession code | 5tbf |

*The highest resolution shell is shown in parentheses.

^†^*R*_merge_ = ∑_h_∑_i_|*I*_i_-〈*I*〉|/∑_h_∑_i_*I*_i_*,* where I_i_ is the observed intensity of the i-th measurement of reflection h, and 〈I〉 is the average intensity of that reflection obtained from multiple observations.

^‡^*R* = ∑||*F_o_*|-|*F_c_*||/∑*|F_o_|,* where F_o_ and F_c_ are the observed and calculated structure factors, respectively, calculated for all data. *R*_free_ was defined in (8).

1. **Gotze M, Pettelkau J, Schaks S, Bosse K, Ihling CH, Krauth F, Fritzsche R, Kuhn U, Sinz A.** 2012. StavroX--a software for analyzing crosslinked products in protein interaction studies. J Am Soc Mass Spectrom **23:**76-87.

2. **Jha JK, Demarre G, Venkova-Canova T, Chattoraj DK.** 2012. Replication regulation of *Vibrio cholerae* chromosome II involves initiator binding to the origin both as monomer and as dimer. Nucleic Acids Res **40:**6026-6038.

3. **Jha JK, Ghirlando R, Chattoraj DK.** 2014. Initiator protein dimerization plays a key role in replication control of *Vibrio cholerae* chromosome 2. Nucleic Acids Res **42:**10538-10549.

4. **Sozhamannan S, Chattoraj DK.** 1993. Heat shock proteins DnaJ, DnaK, and GrpE stimulate P1 plasmid replication by promoting initiator binding to the origin. J Bacteriol **175:**3546-3555.

5. **Pal D, Venkova-Canova T, Srivastava P, Chattoraj DK.** 2005. Multipartite regulation of *rctB*, the replication initiator gene of *Vibrio cholerae* chromosome II. J Bacteriol **187:**7167-7175.

6. **Venkova-Canova T, Srivastava P, Chattoraj DK.** 2006. Transcriptional inactivation of a regulatory site for replication of *Vibrio cholerae* chromosome II. Proc Natl Acad Sci U S A **103:**12051-12056.

7. **Baek JH, Chattoraj DK.** 2014. Chromosome I Controls Chromosome II Replication in *Vibrio cholerae*. PLoS Genet **10:**e1004184.

8. **Brünger AT.** 1992. The free R value: a novel statistical quantity for assessing the accuracy of crystal structures. Nature **355:**472-474.
